# An Overview of Laser Metal Deposition for Cladding: Defect Formation Mechanisms, Defect Suppression Methods and Performance Improvements of Laser-Cladded Layers

**DOI:** 10.3390/ma15165522

**Published:** 2022-08-11

**Authors:** Jian Cheng, Yunhao Xing, Enjie Dong, Linjie Zhao, Henan Liu, Tingyu Chang, Mingjun Chen, Jinghe Wang, Junwen Lu, Jun Wan

**Affiliations:** 1 State Key Laboratory of Robotics and System, Harbin Institute of Technology, Harbin 150001, China; 2 Aircraft Repair & Overhaul Plant, Civil Aviation Flight University of China, Guanghan 618307, China

**Keywords:** laser metal deposition (LMD), grain growth mechanism, stress evolution, defect suppression method, alloy powder

## Abstract

With the development of society and the economy, there is an increasing demand for surface treatment techniques that can efficiently utilize metal materials to obtain good performances in the fields of mechanical engineering and the aerospace industry. The laser metal deposition (LMD) technique for cladding has become a research focus in recent years because of its lower dilution rate, small heat-effect zone and good metallurgical bonding between the coating and substrate. This paper reviews the simulation technology for the melt pool’s grain growth mechanism, temperature and stress distribution that are directly related to defect formation in LMD technology. At the same time, the defect suppression method and the performance improvement method of the cladded layer in LMD technology are introduced. Finally, it is pointed out that the active selection of materials according to the required performance, combined with the controllable processing technology, to form the corresponding microstructure, and finally, to actively realize the expected function, is the future development direction of LMD technology.

## 1. Introduction

### 1.1. Background

In recent years, with the huge demand for wear resistance, corrosion resistance and high-temperature oxidation resistance of key components, surface engineering has become more and more important. Alloys of Mn, Co, Cr, Ni, Al, Ti and other materials are widely used in mechanical engineering, the aerospace industry, biomedical equipment and the electrical industry. It can be more efficient to use these materials by depositing a suitable coating on a substrate [1]. In addition, in the above-mentioned engineering fields, some core components, such as turbine blades, crankshafts, sprockets, etc., have been in service under harsh conditions such as wear, high temperatures and high impact loads for a long time, so they are extremely prone to damage, such as wear, corrosion and fractures, etc. [2,3]. If these parts are not repaired in time, they can only be discarded, causing huge economic losses. Therefore, developing a high-efficiency and resource-saving coating technology to efficiently and accurately repair the surface damage of core components and prolong the service life of these components has become one of the hot research issues in the 21st century.

Traditional coating and repair methods mainly include electroplating, surfacing, tungsten inert gas welding (TIG) and thermal spraying. Although these methods have their own advantages, there are also many problems. For example, tungsten inert gas welding (TIG) has a relatively high energy utilization rate and uses wire as a raw material, which has a low cost, but the cladding layer has poor forming accuracy and a coarse microstructure. Due to the lack of effective protection during the cladding process, the cladding layer is more prone to porosity defects, which limit the application of this technology [3]. With the development of the social economy and the increasing demand for industrial engineering, laser metal deposition (LMD) technology, as a laser surface treatment technology in the field of laser additive manufacturing technology, has the advantages of a low dilution rate, the ability to process difficult-to-machine materials (such as superalloys, titanium alloys, etc.), and a controllable cladding quality. It has gradually become the current mainstream surface treatment technology and the content of frontier scientific research [4,5].

Early concepts of laser additive manufacturing begin circa 1980 with patents by Brown et al., which described layer-wise, additive deposition via combined laser powder (or wire) metallurgy [6,7]. From the 1990s to the beginning of the 21st century, the patents by Lewis et al. [8] and Jeantette et al. [9] promoted the development of this technology by describing systems and methods that use laser beams, controllers and other components to process different powder materials for producing three-dimensional complex objects. Most notably, Hammeke [10] and Buongiorno [11] provided advancements in combined laser/powder delivery mechanisms (e.g., deposition heads), which further increases the feasibility and reliability of LMD technology. Since then, LMD technology has become a research hotspot as a new type of laser additive manufacturing technology being used in mechanical engineering, the aerospace industry, biomedical equipment, military defense and other fields [1,2,3,5].

### 1.2. Laser Metal Deposition Technology

**Figure 1:**
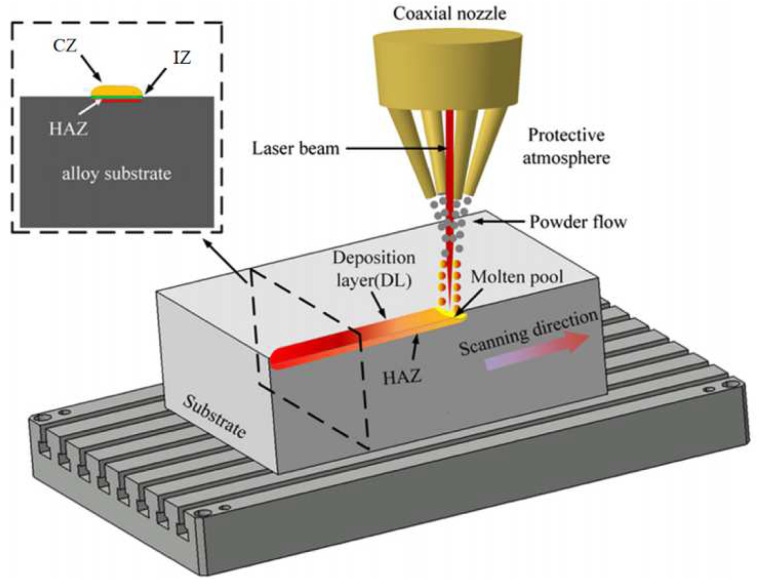
The schematic diagram of the LMD process [24].

Laser Metal Deposition (LMD) processes, also known as Laser Cladding (LC) or Directed Energy Deposition (DED) [12,13,14,15,16], incorporate laser technology, CAD/CAM technology and advanced materials processing technology. The schematic diagram of the LMD process is shown in Figure 1. It uses a laser beam as a high-energy heat source to melt the surface of the substrate and the powdered metal material conveyed by a flowing inert gas (such as argon) through a coaxial nozzle, and a melt pool is generated on the partial surface of the processed workpiece. After laser action, the melt pool quickly solidifies into a metal-cladded layer. When the powder flow and the deposition head (laser beam) move according to the designed scanning strategy, the first cladded layer is deposited on the substrate. Then, the laser deposition head is lifted to a preset distance to deposit the next cladded layer, so that a three-dimensional structure can be formed by multi-layer deposition [17,18]. The cladded layer has excellent physical, chemical and mechanical properties, thereby significantly improving the wear resistance, corrosion resistance and oxidation resistance of the substrate surface. (Fatigue and creep properties are important properties to consider for forming techniques such as SLM rather than coating techniques. Therefore, fatigue and creep performance will not be discussed in this paper). From the surface to the inside, the cladded layer can be divided into three areas: the cladding zone (CZ), the interfacial zone (IZ) and the heat-affected zone (HAZ) [2]. The microstructure of the cladding zone directly determines mechanical properties such as the hardness and wear resistance of the workpiece after LMD processing. The interfacial zone is the bonding area of the cladding powder and the substrate, while the heat-affected zone would affect the microstructure of the substrate or the previous cladded layer through heat treatment and other methods. Generally, the size of the heat-affected zone (HAZ) is reduced by reducing the energy input (such as by reducing the laser power and increasing the scanning speed). The selection and determination of the processing parameters ultimately determines the quality of the processed workpiece, so it is very important to select appropriate processing parameters. Controllable process parameters include laser power (*P*), laser scanning speed (*v*), hatch distance/metal pool overlaps (*d*), powder feed rate/mass deposition rate, spot size (spot diameter *D* or spot area *S*), defocusing amount, etc. [13,19,20,21,22,23].

Compared with other surface treatment technologies, LMD technology has the following advantages: (1) It is cost-effective, and consumes less materials. (2) It applies to a wide range of materials and has strong adaptability to difficult-to-process materials. (3) It can be used to obtain a cladded layer with better properties by designing the material composition, and can process functionally gradient materials. (4) The heat-affected zone and thermal deformation are small. (5) It has a high degree of undercooling, and the melted materials are quickly solidified, so it is easy to obtain a fine-grained structure or produce a metal phase that cannot be obtained under normal conditions. (6) The cladded layer and the substrate can form a metallurgical bond or interface diffusion bond, with high interface strength and few micro-defects. (7) LMD technology uses a laser as its heat source. The laser spot diameter is small, and the scanning speed is fast, so the dilution rate of LMD is low. The low dilution rate leads to the improvement in the surface properties of the substrate (such as wear resistance, corrosion resistance, heat resistance, oxidation resistance and electrical properties). (8) LMD is flexible, with the possibility of automation and integration. (9) LMD has high cleanliness and is environmentally friendly [4,17,19,20,25]. Therefore, LMD technology has attracted widespread attention.

LMD technology can manufacture difficult-to-machine materials such as titanium alloys and superalloys, so LMD has great application potential in the field of repairing and cladding high-value components [14]. When applying LMD technology to repair a workpiece, a subsequent subtractive processing is usually required to meet the required geometric tolerances of the workpiece [13]. Therefore, LMD technology is widely used in various fields such as the aerospace industry, biomedical equipment and mechanical engineering. The development and research of cladding powder materials has always been highly valued by engineers [14]. At present, the commonly used LMD process powders mainly include titanium alloys (such as Ti6Al4V), Ni-based alloy powders (such as Inconel 718 and Ni60), Co-based alloys (such as Stellite 6), Fe-based alloys (such as 316L and H13), etc., and there are a few reports on medium-entropy alloys, ceramics and biological materials [26,27,28,29,30,31,32,33,34,35,36,37,38,39,40,41,42,43].

In view of the advantages and unique role of LMD technology, the research and promotion of this technology has great engineering significance. It should be noted that in the process of processing cladding or deposited layers using LMD technology, different metal-based powders and processing parameters should be selected for different substrates in order to obtain dense and low-defect high-performance cladded layers.

### 1.3. Application

LMD can be used as a promising method for repairing parts because of its small heat-affected zone, metallurgical bonding, small deformation and few defects. At present, the research of repairing mechanical parts based on LMD is mainly focused on the aerospace and nautical ship fields.

In 2010, a geometry-based adaptive toolpath laser powder deposition method was developed to manufacture and repair advanced turbine engine compressors or blisk airfoils [44]. Liu et al. [45] found that laser cladding can be used to improve the corrosion property of Al-based alloy aircraft components. In 2014, Liu et al. [15] successfully repaired casting defects and improperly machined holes in gas turbine engine components using the laser-engineered net shape (LENS) process. In 2015, the Taguchi method was used to optimize the process parameters of multi-layer laser cladding for the repair of steam turbine blades [46]. Paydas et al. [47] used laser cladding technology to deposit Ti-6Al-4V on the processed substrate, simulated the repair process under different conditions and studied the impact of building strategies on the macrostructure, microstructure and hardness. Torims et al. [48] outlined the benefits of LMD technology for in-situ marine crankshaft repairs. In 2016, Liu et al. [49] confirmed that the laser melting deposition could realize the form restoration of groove defects through surface response experiments with different laser powers, scanning speeds and powder feeding speeds. In 2017, Liu et al. [50] found that the Taguchi method can improve the efficiency of laser cladding process parameter optimization, and mechanical components with complex shapes repaired by a laser cladding process show excellent service performance.

In recent years, in the aerospace, vehicle, shipbuilding and other industrial fields, LMD has become more and more important. The use of LMD can realize the remanufacturing of core components, which greatly extends the service life of core components and reduces industrial cost. Therefore, it can be anticipated that the in-depth study and further promotion of LMD will be the future development trend.

However, it should be noted that expanding the application range of LMD technology should be based on understanding the performance improvement of cladding with different alloy powders, while ensuring that the cladded layer is free of defects. Therefore, research on defect formation mechanisms, defect suppression methods and the performance improvement of cladding with different alloy powders in LMD is extremely important. The defect formation mechanism is directly related to the grain growth mechanism in the melt pool and the distribution and evolution of temperature and stress. This work reviews previous efforts on the mechanisms of grain growth in the melt pool, the distribution and evolution of temperature and stress, defect suppression methods and the performance improvement of cladding with different alloy powders in LMD. It is of great engineering significance to promote the application of LMD technology for preparing cladded layer in many fields such as the machinery industry, biomedical equipment, the military industry and national defense, and to further enhance the national economy.

## 2. Mechanisms of Grain Growth in the Melt Pool

In the LMD process, understanding the formation mechanism of defects is a prerequisite for formulating and applying defect suppression methods to ensure the good performance of the cladded layer. This paper reveals the mechanisms of defect formation in the LMD process from three aspects: the mechanism of grain growth in the melt pool, the distribution and evolution of temperature and stress, and the direct cause of the defect formation. In terms of the mechanism of grain growth in the melt pool, when a high-energy laser beam acts on the surface of the substrate and the cladding powder, the substrate and the powder melt to form a melt pool. The complex physical phenomena in the melt pool cause the melt materials to be forced into a flowing state. During the solidification of melt materials in the melt pool, the morphology of the grains is an important factor in determining the structure of the cladded layer. The studies of melt force status, melt flow condition, grain morphology and the influence of melt flows on grain morphology are the basis for further overcoming the defects of the cladded layer by regulating the formation of the microstructure of the cladded layer, which is of great significance.

### 2.1. Melt Force Status

During the LMD process, the melt force status affects the melt flow direction in the melt pool and then affects the microstructure of the cladded layer, which ultimately determines the quality and performance of the cladded layer. Therefore, it is important to analyze the melt force status in the melt pool.

**Figure 2:**
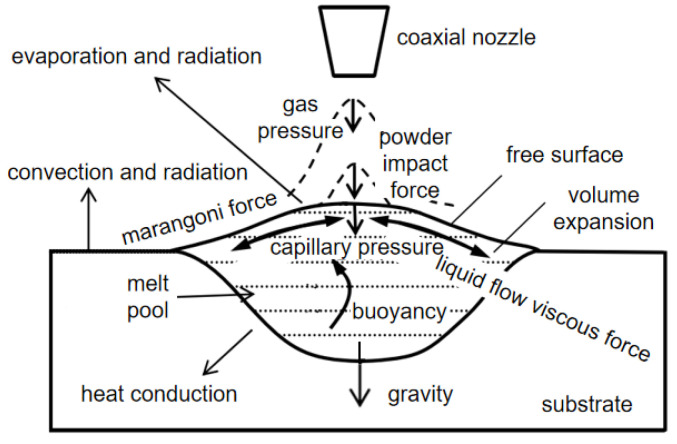
The stress state of the molten pool in the LMD process [51].

Li [51] indicated that in the laser cladding process, there are a variety of complex physical phenomena that work together, such as shielding gas conveying cladding powder, laser and powder interaction, powder and substrate melting, melt flows in the melt pool, heat conduction and so on. Thus, the melt force status in the melt pool is extremely complicated. As shown in Figure 2, in addition to gravity, the forces acting on the melt materials in the melt pool include: capillary pressure, Marangoni force, buoyancy, liquid flow viscous force, internal pressure of the melt pool caused by volume expansion, shielding gas pressure, powder impact force, etc. In the process of laser cladding, all the above-mentioned forces act together on the melting materials in the melt pool, so that the melt materials are in a complex flowing state in the melt pool.

### 2.2. Melt Flow Conditions

The flow of the melt materials in the melt pool is the result of the combined action of various forces, and it is also an important factor influencing the growth and formation of grains in the melt pool. Thus, in order to obtain an idealized grain morphology in cladded layers, it is necessary to understand the melt flow trend in the melt pool and the related factors that affect the melt flow.

**Figure 3:**
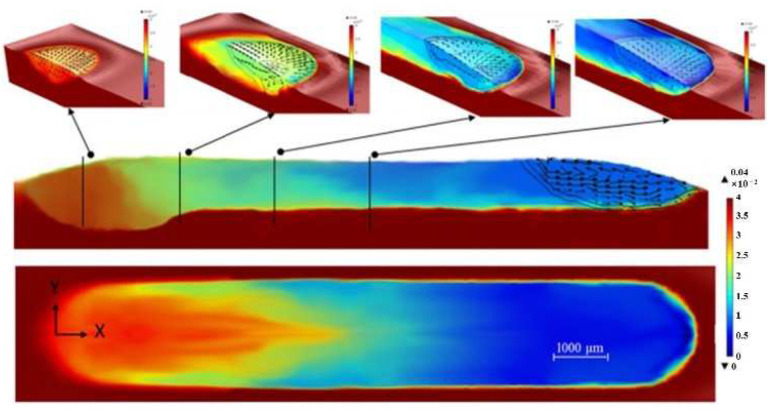
Sulfur concentration profile and melt pool depth distribution in the longitudinal section [54].

**Figure 4:**
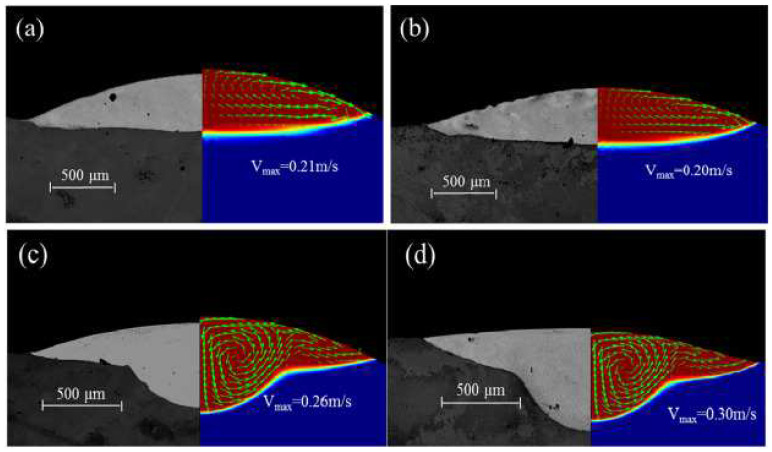
Experimental and calculated geometry of melt pool (y-z plane) under different mass flow rates: (**a**) 6 g/min; (**b**) 4 g/min; (**c**) 2 g/min; (**d**) 0.1 g/min [54].

In 2009, a 3D heat transfer model was developed by Kumar to simulate the LMD process. This model fully considers the complex physical phenomena (such as heat transfer, phase change and the addition of powder particles and fluid flow due to Marangoni–Rayleigh–Benard convection) in the LMD process. It is shown that surface-tension-driven Marangoni–Benard convection is dominant, and buoyancy-driven Rayleigh–Benard convection is insignificant [52]. Heiple et al. [53] proposed a mechanism to reveal the relationship between the surface tension temperature coefficient and the direction of melt flows. They indicated that if the surface tension temperature coefficient is negative (∂σ/∂T<0), the surface tension at the edge of the melt pool would be greater than the center tension of the melt pool. The free surface fluid of the melt pool flows from the center of the upper surface of the melt pool to the edge of the melt pool, and then flows into the bottom of the melt pool along the boundary of the melt pool. Finally, it flows upward near the center of the melt pool to form a reflux, and drives the internal melt materials to flow. On the contrary, if the surface tension temperature coefficient is positive (∂σ/∂T>0), the melt pool would form a clockwise circulation on the left side of the center line of the melt pool, and a counterclockwise circulation on the right side [51]. At this time, the melt pool flows downward to melt the substrate, making the melt pool deeper [54]. Gan et al. [54] studied the impact of the sulfur mass transport and the sulfur mass effects on the Marangoni flow using an improved 3D transient heat transfer and fluid flow numerical model. The results showed that the redistribution of sulfur would affect the state of the Marangoni flow, as seen in Figure 3, as the surface tension temperature coefficient changes from positive to negative as the sulfur mass decreases in the cladded layers. On the left side of the center line of the melt pool, the melt pool changes from a clockwise cycle to a counterclockwise cycle, which eventually causes the depth of the melt pool to change from deep to shallow. The sulfur contents and cross-section geometry of the cladded layers are shown in Figure 4. The sulfur concentration at the top surface of the cladded layers increased 7 times as the mass flow rate decreased from 6 g/min to 0.1 g/min. There are two main flow patterns in the molten pool, that is, an outward flow pattern when the mass flow rate is high (as seen Figure 4a,b) and a predominantly inward pattern when the mass flow rate is low (as seen Figure 4c,d). This is because when the sulfur mass flow rate is high, less of the substrate had been melted, and the concentration of the sulfur at the top of the cladded layers was lower. Negative temperature coefficients of surface tension drive outward flows of melt in the molten pool.

Hu et al. [55] indicated that the melt pool shows an obvious inward flow pattern under the influence of the high sulfur content of T15. In 2018, a high-energy synchrotron micro-radiography technique was used to observe the formation and flow state of melt pools during the laser cladding process by Aucott et al. [56], finding that adjusting the surface-active elements can be used to control the flow state of the internal metal melt. It is worth mentioning that the rare earth elements such as La and Ce are also surface-active elements [57,58,59], which can reduce the surface tension of the molten metal and improve its fluidity, thereby reducing the porosity in the cladded layer and improving the hardness and wear resistance.

Therefore, in the process of LMD for cladding, the Marangoni double-ring vortex, which is affected by the surface tension gradient, can influence the depth of the melt pool by dominating the flow trend of the melt materials. In addition, the surface-active elements such as S, La and Ce can affect the surface tension temperature coefficient, thereby affecting the flow direction of the melt materials, and ultimately affecting the depth and shape of the melt pool.

### 2.3. Grain Morphology

In the LMD technology, the microstructure formed by the solidification of the substrate material and the powder directly affects the mechanical properties of the cladded layer. The cladded layer containing a large number of small equiaxed grains has characteristics of isotropy, good fatigue resistance and good wear resistance. The cladded layer containing thick columnar grains has anisotropy and good high-temperature performance, but is prone to microcracks. Studying the morphology and forming process of grains in the microstructure of the cladded layer is the prerequisite for exploring the mechanisms of crack formation and suppression methods, and it is also the basis for seeking ways to improve the performance of the workpiece, which is of great significance.

**Figure 5:**
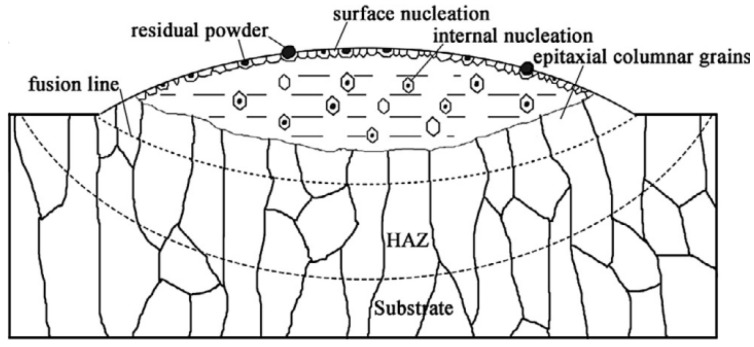
Schematic illustration of the two dominant solidification mechanisms within a local melt pool [22].

The morphology of metal crystals in the cladded layer is mainly equiaxed grain and columnar grain. Wang et al. [22] revealed the grain morphology evolution behaviors of laser-deposited titanium alloy components via studying the influence of the mass deposition rate on the structure of the cladded layer during the LMD layer-by-layer cladding process. They pointed out that there are two main solidification mechanisms in the LMD process, i.e., as shown in Figure 5, the upper part of the cladded layer is mainly composed of small-sized equiaxed crystals, and the lower part of the cladded layer is mainly composed of coarse columnar crystals produced by grain epitaxy; these two growth mechanisms compete with each other, which together determine the grain morphology of the cladded layer. In addition, increasing the mass deposition rate would cause the expansion of the equiaxed grain area in the cladded layer.

For the growth of columnar grains at the bottom of the melt pool during LMD, Henry et al. [60] pointed out that dendrites usually grow along the direction perpendicular to the substrate and closest to the direction of the heat flow <001>. In 2016, Zhang et al. [61] pointed out that in the process of melt materials’ solidification, most of the grains tend to grow along the <001> direction and form a grain boundaries misorientation angle of about 2°. However, in the bottom area of the melt pool, since the solid–liquid interface is arc-shaped, the dendrite growth is not strictly perpendicular to the surface of the substrate. This would cause the bottom grain boundary to be more disordered, and some grain sizes are smaller than other parts of the deposited layer. The hardness of different areas of the cladded layer is different due to the changes in eutectic morphology, grain morphology distribution, grain boundaries misorientation and the precipitation of a small amount of strengthening phase in different areas.

**Figure 6:**
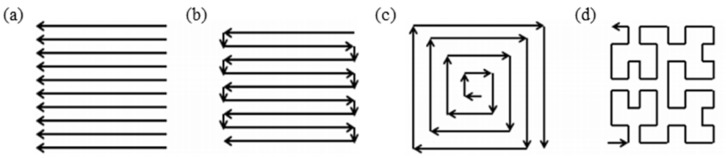
Different deposition patterns: (**a**) raster; (**b**) bi-directional; (**c**) offset-out; (**d**) fractal [14].

When performing multi-layer laser cladding, there is a remelting phenomenon between two adjacent cladded layers, which affects the growth of grains. Thijs et al. [62] indicated that in the laser additive manufacturing technology, the laser scanning strategies, which include raster, bi-directional, offset-out and fractal [63] (Figure 6), can affect the grain growth direction by affecting the local heat transfer conditions, and ultimately can affect the microstructure. In the case of layer-by-layer cladding, due to the partial remelting of the previous layer, the columnar grains can grow further.

Therefore, in the melt pool of LMD, the crystal grain morphology is dominated by equiaxed grains on the surface of the melt pool and columnar grains at the bottom. The columnar grains grow at the bottom of the melt pool along the direction perpendicular to the solid–liquid interface and closest to the heat flow. When the multi-layer laser cladding is processed, the equiaxed grains on the upper part of the melt pool in the previous cladded layer may be remelted, so that the columnar grains at the bottom of the melt pool can grow. It is worth noting that during the LMD process, the laser scanning strategy can affect the direction of grain growth by affecting the local heat transfer conditions, and ultimately affect the structure of the cladded layer.

### 2.4. Influence of Melt Flow on Grain Morphology

Since the melt materials are in a flowing state and the columnar grains at the bottom of the melt pool grow along the direction closest to the heat flow, the melt flow is directly related to the morphology of the crystal grains. Studying the influence of the melt flow on grain formation and growth is a prerequisite for regulating the formation of the microstructure of the cladded layer, and it is of great significance to improving the performance of the cladded layer.

**Figure 7:**
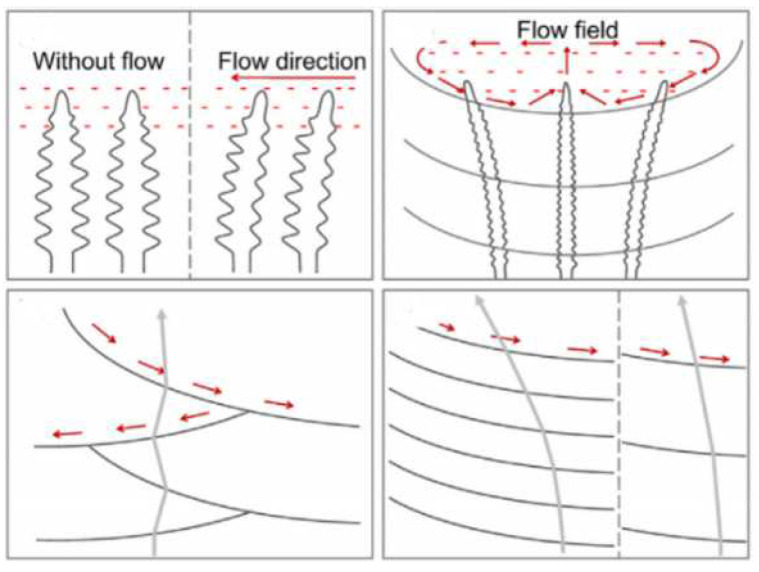
Schematic diagram of the deflection trend of the LMD process [65].

In 2001, Canalis et al. [64] found that the flow of the melt pool contributes to dendrite fragmentation and the transport of dendrite arms; these dendrite arms and unmelted powder particles create a large number of nucleation sites for the solidification of the melt in the melt pool. Wang et al. [65] prepared Ni-based alloys on a single crystal substrate by LMD. They found that the flow field in the melt pool is an important factor that causes the deflection of dendrite growth during the layer-by-layer deposition process. As shown in Figure 7, dendrites grow along the direction of the melt flow. In 2016, Chen et al. [66] studied the influence of the laser input angle on the dendritic microstructure, crystal orientation and the heat-affected zone (HAZ) liquation cracking tendency of Inconel 718 deposited on a polycrystalline substrate. They found that laser input angle can affect the growth of second dendrite arms, because adjusting the laser input angle can change the lateral temperature gradient, while at the same time, also making dendrites grow from [001] to [100] and can inhibit the formation of cracks in the heat-affected zone.

Similar grain morphologies were also found in LMD-fabricated Ti-based alloys [14,22,28]. There are few reports on the fabrication of Cu-based alloys by LMD technology. This is because the Cu element is usually present as a non-major element in the alloys (such as Al–Cu alloy, Al–Zn–Mg–Cu alloy) commonly used in LMD technology, so Cu-based alloys are not discussed here. Therefore, the melt flow in the melt pool has two main effects on the formation and growth of grains. On the one hand, the melt flow can promote the formation of equiaxed grains by breaking dendrites. On the other hand, the melt flow can affect the growth of columnar grains at the bottom of the melt pool by changing the direction of the heat flow. It should be noted that the grain morphologies in the LMD process are the result of a combination of factors such as the temperature conditions and melt flow in the molten pool.

### 2.5. Control the Cladding Microstructure by Coupling Physical Fields

**Figure 8:**
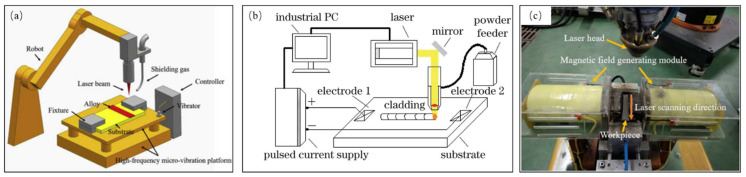
Schematics diagram and equipment diagram for coupling physical fields: (**a**) the schematic diagram of high-frequency micro-vibration coupling fields; (**b**) the schematic diagram of electric coupling fields; (**c**) the equipment diagram of magnetic coupling fields.

In view of the fact that the melt flow in the melt pool has a direct impact on the formation and growth of crystal grains, the LMD process can be coupled with different physical fields such as high-frequency micro-vibration, ultrasonic vibration, and electric and magnetic fields to affect the melt flow in the melt pool; the schematics diagram and equipment diagram for coupling physical fields are shown in Figure 8. So, the formation and growth of crystal grains would be affected to achieve the purpose of regulating the microstructure of the cladded layer.

**Figure 9:**
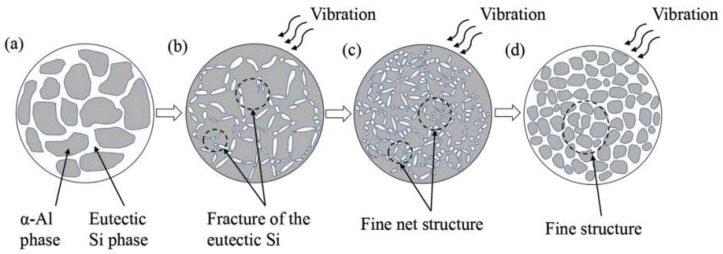
Evolutionary process of the fine microstructure obtained under high-frequency micro-vibrations: (**a**) molten liquid phase before solidification; (**b**) fracture of the eutectic Si under high-frequency micro-vibrations; (**c**) fine eutectic Si obtained by high-frequency micro-vibrations; (**d**) fine-grained and compact structure in the alloy [67].

In 2019, TiC/AlSi10Mg composite cladded layers were successfully fabricated on high-frequency microvibration platforms using the LMD process. During the solidification process, the long eutectic Si particles were broken by high-frequency vibrations (as seen in Figure 9b) and distributed uniformly with the flow of the melt (as seen in Figure 9c). These broken eutectic Si particles serve as nucleation sites to form a fine net structure. The net structure and the α-Al phase are closely combined to form a dense microstructure (as seen in Figure 9d) [67].

**Figure 10:**
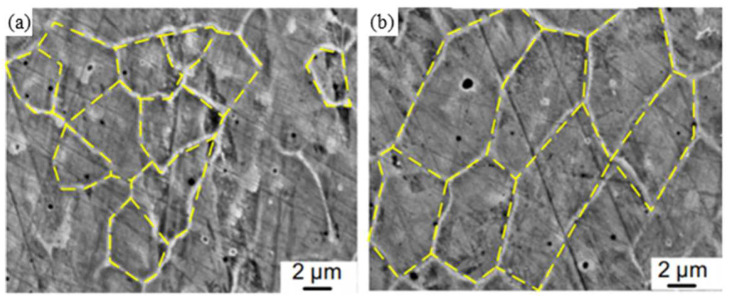
Effects of ultrasonic vibrations on the grain size of AISI 630 thin walls fabricated by LENS: (**a**) with ultrasonic vibrations; (**b**) without ultrasonic vibrations [68].

In 2017, Cong et al. [68] combined ultrasonic vibrations with the laser-engineered net shape (LENS) process, finding that ultrasonic vibrations will generate periodic positive and negative pressure changes in the molten pool, thereby promoting the flow of the melt. It can be seen from Figure 10a,b that the grain size in the UV-A LENS process is finer than the grain in the thin walls fabricated by LENS without ultrasonic vibrations. This is due to the acoustic streaming and cavitation effects brought by ultrasonic vibrations, which generate instantaneous impact stress and temperature fluctuations in the melt pool, making the solidification front unstable. The broken grains flow back into the molten pool through the melt flow and become new nucleation sites, so the microstructure of the cladded layer changes from columnar grains to equiaxed grains [69].

Xie [70] introduced a pulse current into the laser cladding process and indicated that when the current passes through the melt, there would be an electromigration effect, Joule heating effect, Peltier effect, skin effect and hysteresis constriction effect. The introduction of a pulsed current can increase the degree of supercooling during metal solidification, thereby increase the nucleation rate and promote grain refinement [71]. In addition, different current densities in different regions of the metal melt would cause different shrinkage forces, resulting in a difference in the internal flow rate of the melt and shearing stress. If the shearing stress is large enough, the dendrites will be broken into nucleation sites for equiaxed grains, thereby promoting grain refinement [72].

**Figure 11:**
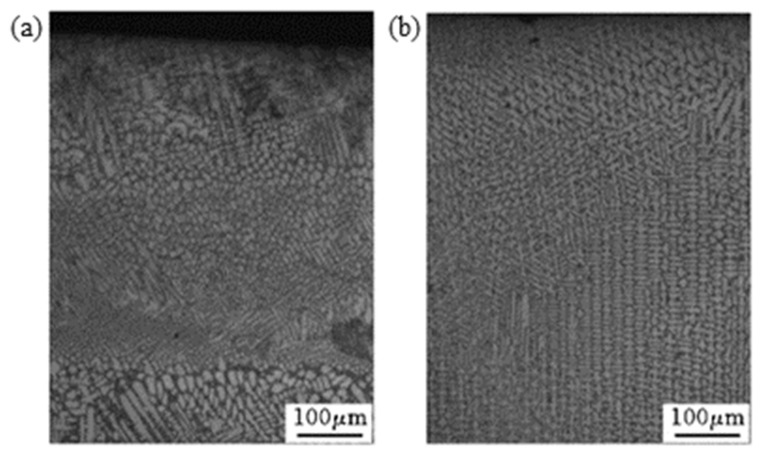
Effects of alternating magnetic fields on the grain size in Fe-based cladded layers fabricated by LMD (the laser power is 3 kw and the scanning speed is 350 mm/min): (**a**) without alternating magnetic fields; (**b**) with alternating magnetic fields [75].

In the research on the influence of magnetic fields on the solidification process of LMD, Li et al. [73] studied the effects of strong magnetic fields on the columnar-to-equiaxed transition (CET) during alloy solidification. They indicated that in the melt pool, the magnetic field interacts with the current generated by the flow of particles and the thermoelectric current generated by the thermoelectric effect to produce the Lorentz force and the thermoelectric magnetic force, respectively. The Lorentz force, thermoelectric magnetic force and magnetization force owing to the magnetic anisotropy of the dendrite work together on cells/dendrites and equiaxed grains, causing the cells/dendrites to break and driving the equiaxed grains to rotate to further destroy the cells/dendrites. Thus, applying a strong magnetic field during the solidification of the alloy would cause the fragmentation of cells/dendrites and the columnar-to-equiaxed transition. Zhao [74] applied the alternating magnetic field which is generated by a self-designed magnetic field device in the laser cladding process of Fe-based alloys. It was indicated that the electromagnetic stirring technology is based on two basic principles: first, Faraday’s law of electromagnetic induction, that is, the conductive liquid generates an induced current when cutting the magnetic line of induction in a magnetic field; second, the charged body is subjected to an electromagnetic force in the magnetic field. Fu et al. [75] prepared a high-hardness Fe-based alloy (0.15 wt%C, 2.4 wt%B, 30 wt%Cr, Bal. Fe) cladding layer on a Q235A substrate, and the experimental results are shown in Figure 11. Compared with the structure of the cladded layer under the action of no magnetic field (Figure 11a), the equiaxed grain area in the structure of the cladded layer under the action of a magnetic field (Figure 11b) is significantly enlarged.

The coupling of physical fields and its effects are summarized in Table 1. As seen in Table 1, coupling LMD technology with high-frequency micro-vibrations, ultrasonic vibrations, electric fields, magnetic fields and other physical fields is a feasible method to control the microstructure of the cladded layer. High-frequency micro-vibrations can increase the strength of the melt flow and break long strip dendrites. The ultrasonic vibrations promote the formation of a large number of equiaxed grains by acoustic streaming, cavitation and increasing the energy in the melt pool to increase the thermal gradient. The introduction of a current during the LMD process would cause a series of positive effects such as the electromigration effect, Joule heating effect, Peltier effect and so on, which will lead to grain refinement. The application of a strong magnetic field in the molten zone would generate a Lorentz force, thermoelectric magnetic force and magnetization force, which cause cells/dendrites to break and then the grains of the cladded layer to be refined.

In addition to the physical field-coupling technology, in recent years, cryogenic quenching to improve the performance of the cladding layer has begun to attract researchers’ attention. Zhang et al. [76] quenched the deposited IN718-cladded layer in liquid nitrogen: the increased cooling rate reduced the segregation of niobium, and the aged hardness increased by 4%. In the research on the cryogenic quenching of Co-based [77] and Fe-based [78] alloys, the cryogenic initial temperature of the substrate dramatically reduced the clad dilution compared to a room temperature substrate. The hardness increased because of the reduction in the secondary dendrite arm’s spacing.

In summary, in the LMD process for cladding, on the one hand, the melt materials in the melt pool are in a flowing state under the action of many forces, and the surface-active elements such as S, La and Ce can affect the surface tension temperature coefficient of the melt materials in the melt pool, thereby affecting the flow direction of the melt driven by the Marangoni double-ring vortex, and ultimately affecting the depth and shape of the melt pool. On the other hand, the grain morphology in the melt pool is dominated by equiaxed grains on the surface of the melt pool and columnar grains at the bottom. The number of equiaxed grains formed at the top of the melt pool is proportional to the nuclei density, and columnar grains grow at the bottom of the melt pool perpendicular to the solid–liquid interface and along the direction closest to the heat flow. The nuclei density affecting the distribution of equiaxed grains is related to two phenomena: part of the cladding powder is unmelted, and the melt flow breaks dendrites in the melt pool. The laser scanning strategy, the laser input angle and other factors can affect the direction of the heat flow in the melt pool to affect the direction of grain growth, and ultimately can affect the microstructure of the cladded layer. In addition, when the solid–liquid interface at the bottom of the melt pool is irregular, the dendrite growth direction at the bottom of the melt pool would not be perpendicular to the substrate, resulting in smaller grain sizes at the bottom of the melt pool and more disordered grain boundaries. When performing multi-layer laser cladding processing, the equiaxed grains on the upper part of the melt pool in the previous cladded layer can be remelted by changing the process parameters, so that the columnar grains at the bottom of the melt pool can grow further. Thus, the melt flow in the melt pool has two main effects on the formation and growth of grains. On the one hand, the melt flow can promote the formation of equiaxed grains by breaking dendrites. On the other hand, the melt flow can affect the growth of columnar grains at the bottom of the melt pool by changing the direction of the heat flow. When the LMD technology is coupled with high-frequency micro-vibrations, ultrasonic vibrations, electric fields, magnetic fields and other physical fields, different physical fields directly or indirectly affect the melt flow and break the crystal grains in the melt pool to cause the expansion of the equiaxed grain area in the cladded layer, thereby realizing the control of the microstructure of the cladded layer. The study of the influence of the melt flow on grain morphology is the basis for studying the control of the microstructure of the cladded layer by coupling different physical fields. Only by understanding the grain growth mechanism in the melt pool can we further explore the direct causes of defects and formulate methods to suppress cladding defects from the perspective of the microstructure, so as to obtain a cladded layer with excellent performance.

## 3. Distribution and Evolution of Temperature and Stress

In the LMD process, the mechanism of defect formation is not only related to the grain growth mechanism in the melt pool, but also to the evolution and distribution of stress during the laser processing process. The evolution and distribution of stress are directly related to the evolution and distribution of the temperature. The evolution and distribution of temperature affect not only the microstructure of the cladded layer but also the evolution and distribution of stress. The evolution and distribution of stress determine the formation of cracks in the cladded layer by affecting the magnitude of the residual stress. Therefore, in order to reveal the defect formation mechanism, formulate defect suppression methods and obtain an excellent performing cladded layer, it is necessary to study the evolution and distribution of the temperature and stress during the LMD process.

### 3.1. Distribution and Evolution of Temperature

In the LMD process, the high-temperature area near the heat source has a very obvious temperature gradient, and the cladded layer being processed by the laser would affect the thermal history of the nearby cladded layer and then affect the formation of the cladded layer microstructure in the nearby area.

**Figure 12:**
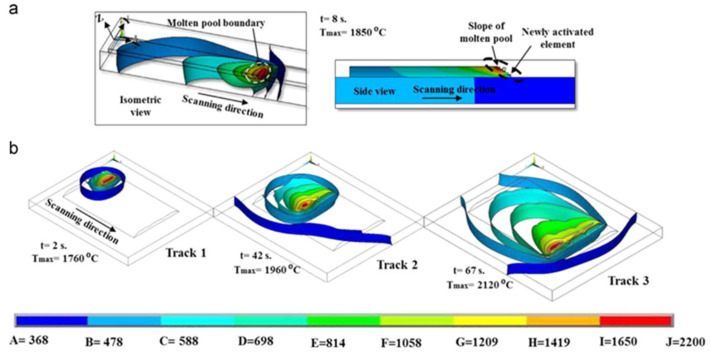
Temperature gradient during the laser cladding: (**a**) single-track laser cladding; (**b**) multi-track laser cladding [4].

In 2014, a three-dimensional (3D) transient uncoupled thermoelastic-plastic model has been proposed. This model was used to analyze the thermal process and thermally-induced residual stress of the laser cladding process. As shown in Figure 12a, a comet-tail profile-like molten area was observed in single-track laser cladding simulation results. In the multi-track laser cladding, the temperature distribution skews along the former deposited tracks [4].

**Figure 13:**
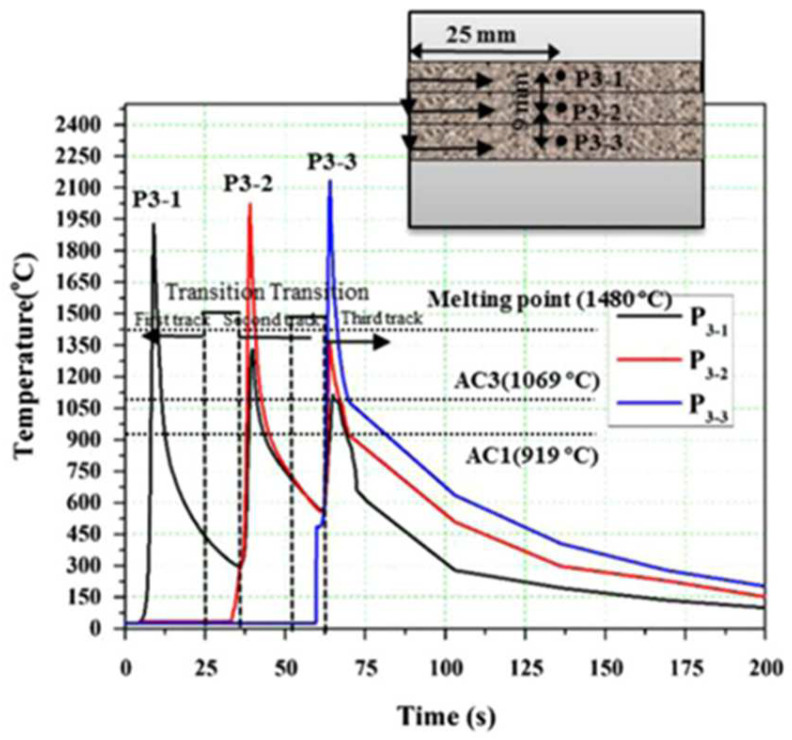
AISI H13 steel multi-track laser cladding temperature evolution versus cladding time (the laser power is 3800 w, and the scanning speed is 300 mm/min) [4].

In terms of heat transfer, during the laser cladding process, the substrate near the cladding area is preheated by heat conduction. When the laser scans to this point, the temperature reaches the maximum value. After the laser leaves this point, the workpiece dissipates heat through the substrate heat conduction, cladded layer surface and air convection and heat radiation, so the temperature drops sharply [79,80]. The temperature of this point would rise and fall again when performing the next cladding tracks. Figure 13 shows the temperature evolution at three different monitoring locations during multi-pass cladding of AISI H13 steel with a laser power of 3800 w and a scanning speed of 300 mm/min. In addition, there would be a thermal cycling effect characterized by localized temperature field disturbances with a sharp temperature increase and decrease in the layer-by-layer laser cladding process. This effect causes heat treatment or solid-state phase transitions, leading to microstructure evolution and thermal–mechanical interactions.

The simulation analysis of the LMD process shows that the temperature gradient near the laser heat source is large. The previous cladding track not only preheats the latter cladding track, but also cyclically heats the adjacent cladding tracks of the previous layer. Therefore, the temperature evolution process of the overlap area and the adjacent area of the LMD-cladded layer would lead to heat treatment or solid-state phase transitions, which causes microstructure evolution and thermal–mechanical interactions, such as thermal warpage and residual stress formation. In addition, in the process of multi-layer (long-time heating) or large-area laser cladding, the warpage or deformation of the substrate due to thermal stress on the substrate cannot be ignored. Usually, this adverse effect can be improved by adding water cooling channels under the substrate or adopting intermittent cladding.

### 3.2. Distribution and Evolution of Stress

The analysis of the stress composition, distribution and evolution of the cladded layer prepared by LMD technology is the basis for further exploring the causes of cracks perpendicular to the scanning direction in the cladded layer.

In the LMD process, on the one hand, due to the difference between the thermal and physical properties of the cladding material and the substrate, such as the thermal expansion coefficient, thermal conductivity, etc., the temperature distribution in the cladding area would be uneven, which will affect the generation and distribution of thermal stress. At the same time, due to the large temperature gradient, there will be some phase changes in the overlap zone and the heat-affected zone, resulting in compressive or tensile stress. On the other hand, plastic strain, elastic strain and thermal strain mainly occur in the cladding area. Among them, the change in plastic strain is not significant. The elastic strain firstly drops to a negative value, and then increases to a positive value during the cooling process and remains unchanged. The thermal strain has a peak when the laser beam passes, and then gradually decreases as the melt materials cool down [4]. The residual stress refers to the stress that an object has in a state of mechanical and thermal equilibrium in the absence of an external force. Therefore, the residual stress produced by the cladding process is mainly composed of compression or tensile stress and thermal stress. The residual strain is composed of elastic strain and thermal strain.

For the relationship between stress and strain, Zhang et al. [81] used the finite element method to simulate the distributions of the temperature field and stress field in the LMD process. It was indicated that because the length of the substrate along the laser scanning direction is greater than the width along the direction perpendicular to the laser scanning direction and the thickness of the substrate, the workpiece undergoes the greatest deformation resistance along the laser scanning direction after cladding. The strain distribution in the three directions near the junction of the cladded layer and the substrate is as follows: the strain along the thickness of the substrate is greater than the strain in the transverse direction (perpendicular to the scanning direction) and greater than the strain in the longitudinal direction (scanning direction). Correspondingly, the residual stress distribution in these three directions is as follows: the residual stress in the thickness direction of the substrate is smaller than the transverse residual stress and smaller than the longitudinal residual stress [77]. Thus, the maximum residual tensile stress along the scanning direction is the main cause of cracks in the cladded layer perpendicular to the scanning direction.

**Figure 14:**
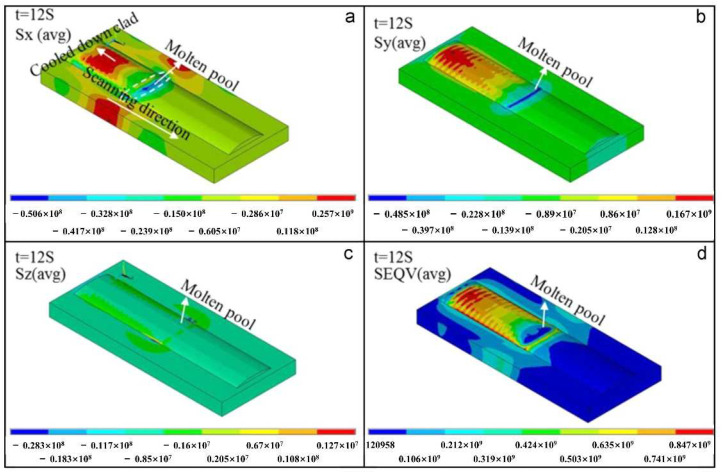
3D-modeled contours of stress components in single-track laser cladding: (**a**) longitudinal stress SX; (**b**) transverse stress SY; (**c**) along-thickness stress SZ; (**d**) Von Mises equivalent stress SEQV [4].

When performing single-track or multi-track laser cladding, the stress evolution process of the cladded layer is slightly different, but the stress distribution is roughly the same. For single-track laser cladding, Farahmand et al. [4] pointed out that according to the Von Mises stress distribution, the high stress concentration of laser cladding mainly exists in the cladding zone (CZ) and the heat-affected zone (HAZ). The cladding zone (CZ) and the interfacial zone (IZ) are high tensile stress zones, and the heat-affected zone (HAZ) is a compressive stress zone [82]. The generation of the residual compressive stress of the substrate is caused by the substrate material undergoing a phase change (such as martensite, etc.) when the laser heat source acts, thereby generating additional compressive stress. At the same time, due to the mechanical balance, the compressive stress of the substrate also increases the tensile stress in the cladded layer. Figure 14 shows the stress distribution diagram of a single-track laser cladding. In the case of multi-track laser cladding, the residual stress of the previous cladding track deposited is relatively low due to the cyclic heating and cooling of the subsequent cladding track [83]. The stress of each cladding track evolves into a repeated cyclic stress of compression–tension–compression–tension until the cladding is over. The stress concentration at the interface between the cladded layer and the substrate is relatively high, and it is more sensitive to the formation of cracks. The interfacial zone (IZ) has a high degree of stress concentration and is more sensitive to crack formation.

**Figure 15:**
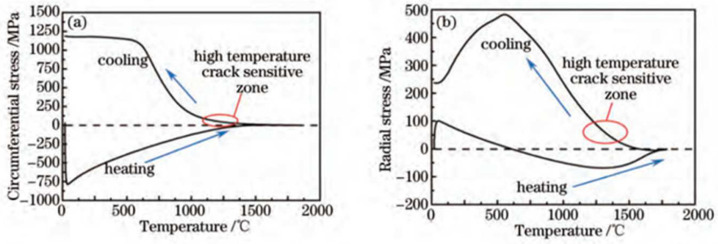
Temperature–circumferential of Inconel 718 alloy: (**a**) and temperature–radial; (**b**) stress curve in the cladding metal melting and solidification process [84].

As the melt materials in the melt pool melt and solidify, the stress in the cladding zone (CZ) is constantly changing. In 2015, Dai et al. [84] used the finite element method to simulate the temperature field and stress field of the ring laser cladding process on Inconel 718 Ni-based alloys. They found that during the heating process, the cladding metal is subjected to certain circumferential (scanning direction) and radial (perpendicular to the scanning direction) compressive stress within a certain temperature range due to heating. When the temperature reaches a certain value, the stress on the cladding metal almost becomes zero. When the cladding metal is cooled and solidified, the stress on it at the beginning is very small. When the temperature reaches a certain value, the stress in the circumferential and radial directions begins to appear as tensile stress. If the tensile stress of the cladding metal is greater than its plasticity, the cracks may occur. The temperature–stress relationship curve of the laser cladding Inconel718 alloy stress evolution process is shown in Figure 15.

It is worth mentioning that in addition to the thermal history and phase transition of the cladded layer, the cause of residual stress is also related to the thermal conductivity, thermal expansion coefficient, Young’s modulus and yield stress of the substrate material and alloy powder, as well as the geometric shape of the workpiece, the processing parameters, the scanning strategy and other factors. Thus, selecting a powder material with a thermal expansion coefficient similar to that of the substrate, selecting a suitable LMD scanning strategy, preheating the substrate, reducing the laser power and increasing the laser scanning speed can reduce the residual stress of the cladded layer [2,4,82]. One of the important reasons for the stress in the laser-cladded layer is the large difference between the thermal expansion coefficient of the cladding alloy and the substrate. So, a reasonable choice of alloy powder with a thermal expansion coefficient similar to the substrate is a way to reduce the residual stress of the cladded layer and reduce its crack sensitivity. Dai et al. [85] used finite element analysis to study the effect of the laser scanning strategy on the residual stress and deformation of the cladded layer. They found that by using the offset-out scanning strategy (Figure 6c), the residual stress can be reduced to one third of the residual stress produced by the bi-directional scanning strategy (Figure 6b). In 2017, Yang et al. [86] indicated that laser cladding can reduce the residual stress between the cladded layer and the copper substrate by preheating, which can also suppress the occurrence of cracks.

Therefore, In the LMD process, the residual stress is mainly composed of compressive or tensile stress and thermal stress, while the residual strain is composed of elastic strain and thermal strain. The relationship between stress and strain is affected by the structure of the substrate. Along the direction where the deformation resistance of the substrate is greatest, the strain is the smallest, the residual stress is the largest and the cracks are most likely to occur. The cladding zone (CZ) and the interfacial zone (IZ) are tensile stress zones, and the heat-affected zone (HAZ) is a compressive stress zone. The interfacial zone (IZ) stress concentration is higher, and it is more sensitive to crack formation. In multi-track laser cladding, the previous cladding track has relatively low residual stress due to the cyclic heating and cooling effects of the subsequent cladding track. During laser processing, the cladding metal is firstly subjected to compressive stress. Then, the stress becomes zero as the temperature rises. Finally, as the molten metal cools and solidifies, tensile stress appears in the scanning direction and the direction perpendicular to the scanning direction. If the tensile stress is greater than the plasticity of the cladding metal, cracks will occur. In addition, the generation of residual stress is also related to the thermal conductivity, thermal expansion coefficient, Young’s modulu, and yield stress of the substrate material and alloy powder, as well as the part’s geometry, processing parameters, scanning strategy and other factors. Selecting powder materials with a thermal expansion coefficient similar to the substrate, selecting a suitable LMD scanning strategy, preheating the substrate, reducing the laser power and increasing the laser scanning speed can reduce the residual stress of the cladded layer, thereby reducing the crack sensitivity of the cladded layer.

In summary, in addition to the obvious temperature gradient near the heat source, the difference between the thermal and physical properties of the cladding material and the substrate, such as the thermal expansion coefficient, thermal conductivity, etc., as well as the processing parameters and scanning strategy can cause an uneven temperature distribution in the laser cladding area, thereby affecting the generation and distribution of thermal stress. Moreover, different areas of the cladded layer have different thermal histories directly related to the temperature field, so there will be different phase transitions in the cladding zone (CZ), the interfacial zone (IZ), HAZ and overlap areas, resulting in tensile or compressive stress. The thermal stress and tensile or compressive stress together constitute residual stress. The relationship between stress and strain is affected by the structure of the substrate. Along the direction where the deformation resistance of the substrate is greatest, the strain is the smallest, the residual stress is the largest and the cracks are most likely to occur. There are several potential ways to reduce the residual stress of the cladded layer: selecting powder materials with a thermal expansion coefficient similar to the substrate, selecting a suitable LMD scanning strategy, preheating the substrate and adjusting the process parameters to reduce the laser energy density. Therefore, studying the distribution and evolution of temperature and stress can further reveal the formation mechanism and hence facilitate the development of defect suppression methods for obtaining well-performing cladded layers.

## 4. Defect Formation and Suppression

The formation mechanism of defects in LMD technology can be revealed by understanding the mechanisms of grain growth in the melt pool, as well as the distribution and evolution of temperature and stress. However, to ensure the quality and performance of the cladded layer, it is necessary to understand the direct causes of defects and to develop defect suppression methods. The main defects of workpieces processed by LMD are pores and cracks. Studying the direct causes of cracks and pores and proposing corresponding methods is the basis for further improving the performance of cladded layers with different alloy powders, which are of great significance for optimizing the quality of cladded layers and promoting laser cladding technology.

### 4.1. Causes of Pore Formation

Porosity is a key factor for some problems such as stress concentration, performance degradation and so on [87]. Thus, in order to prepare cladded layers with good performances, it is necessary to explore the direct causes of pore formation. Only by understanding the reasons for pore formation can we further develop methods to suppress pores.

**Figure 16:**
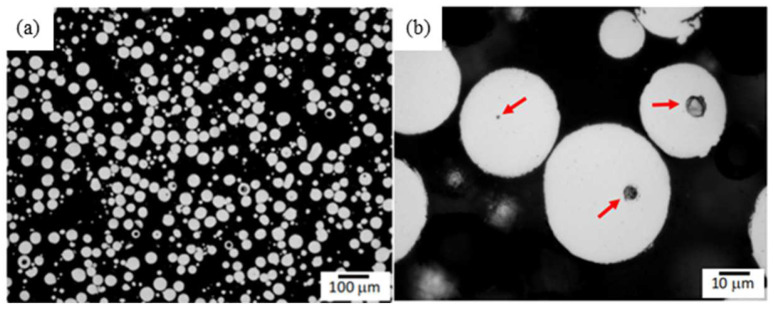
Light optical micrographs of as-polished cross sections of raw 316L SS powders: (**a**) Low magnification showing the particle size distribution and inherent gas pores inside the powder particles; (**b**) Individual powder particles show small spherical gas pores (marked by arrows) [87].

In 2019, Choo et al. [87] indicated that the porosity in the powder itself is one of the reasons that the laser additive manufacturing workpiece contains pores. Figure 16 is the light optical micrograph of the as-polished cross-section of the 316L Stainless Steel powder used in the work. These small spherical pores in powders are considered to be caused by the argon staying in the powder particles and not escaping during the process of manufacturing the metal powder, eventually forming hollow powders [88,89]. In the additive manufacturing process, the gas in the hollow powder enters the melt pool and is trapped during the solidification process, and finally remains in the additive manufactured workpiece [38,89].

In 2019, Zhang et al. [90] pointed out in comparative experiments of laser welding and tungsten inert gas welding (TIG) between laser selective cladding and cast AlSi10Mg that the oxygen content in the laser selective cladding AlSi10Mg plate is much higher than that in the as-cast AlSi10Mg plate. Oxides easily absorb water and protective gas in the air and induce reaction (1) under the laser action. The solubility of hydrogen in liquid aluminum can reach 0.7 mL/(100 mg), while that in solid aluminum is only 0.036 mL/(100 mg). So, the hydrogen generated by aluminum and water under the laser action is precipitated during the process of aluminum alloy changing from liquid to solid, resulting in the generation of pores. As for the oxide Al_2_O_3_, it can be seen as an oxide film on the powder or substrate. The oxide film usually causes irregular pores of about tens of microns in the cladded layer [91]. However, when Liao et al. [92] studied the mechanism of alumina loss in laser selective cladding of Al_2_O_3_–AlSi10Mg composites, it was found that excessively high temperatures in the melt pool during cladding will cause the reduction reaction of aluminum to alumina in the melt pool (2), resulting in pores in the cladded layer.

2Al + 3H_2_O = Al_2_O_3_ + H_2_↑ (1)

4Al + Al_2_O_3_ = 3Al_2_O↑ (2)

**Figure 17:**
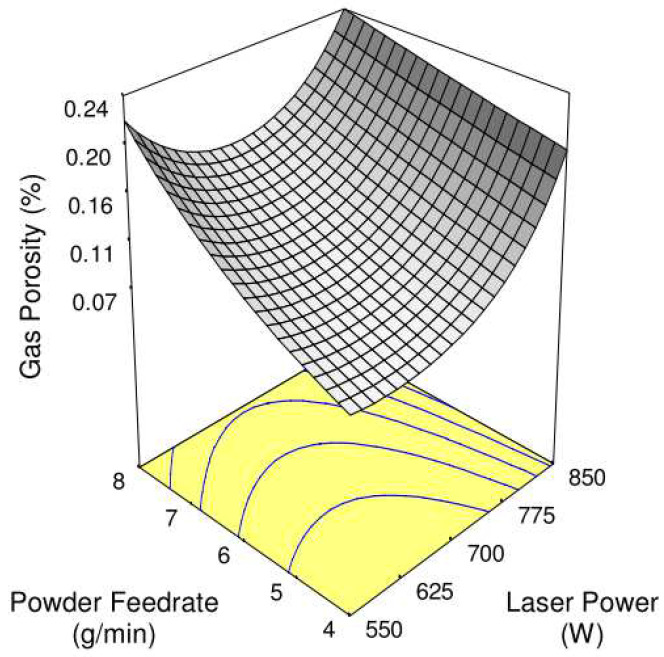
The influence of the laser power and powder feeding rate on porosity in the LMD process [23].

The LMD processing parameters can directly or indirectly affect the melt flow in the melt pool so as to affect pore formation. Ng et al. [23] discussed the relationship between LMD process parameters and porosity in studying the formation of pores and bubble retention during the LMD process. As shown in Figure 17, as the laser power increases, the porosity first decreases and then increases. It was found that an increase in laser power will increase the input heat and reduce the solidification rate, allowing bubbles to escape before the melt materials in the melt pool solidify, and it will also reduce the pores caused by insufficient melted powder [93]. However, if the laser power is too high, the melt flow in the melt pool will be more violent and will aggravate the powder, trapping the shielding gas and bringing it into the melt pool to form pores, which is also the reason for the increase in the porosity caused by the increase in the powder feeding rate. It is worth mentioning that the effect of the Marangoni flow on driving the flow of bubbles is 5 times the effect of the floating bubbles themselves, so the Marangoni flow will cause bubbles to deposit at the bottom of the melt pool or promote bubbles to coalesce when the melt flows, causing bubbles to aggregate and produce large pores. Kumar et al. [21] pointed out that in the laser additive manufacturing of Inconel 718, choosing the right hatch distance can help reduce the porosity between two adjacent cladded layers through remelting. However, in 2019, Langebeck et al. [94] found that the oxide layer on the surface of the aluminum alloy will produce a chemical reaction during the LMD process, and the gas produced in this chemical reaction causes the pore volume to increase as the hatch distance increases.

In summary, the main reasons for the formation of pores in the LMD process are that (1) the powder itself contains pores, (2) gas generated by some chemical reactions during the laser processing process leads to the generation of pores, (3) the improper selection of process parameters causes the bubbles in the melt pool to fail to escape before the melt materials solidify, and (4) the protective gas is involved in the melt materials to form pores during the melt material solidification process.

### 4.2. Methods of Pore Suppression

Understanding the direct causes of pore formation, it has become a current research hotspot to develop corresponding pore suppression methods. In LMD for cladding, the use of effective methods to suppress pores can greatly reduce the stress concentration and improve the performance of the workpiece, which is of great significance for further research on the performance of cladded layers prepared by various powders.

Firstly, the low-porosity powder can be used to obtain a low-porosity cladded layer. Zhao et al. [16] used LRF technology to deposit Inconel 718 powders. They found that the powder material produced by plasma rotating electrode preparation (PREP) has a lower porosity compared with the gas atomized powder.

Secondly, the porosity of the cladded layer can also be reduced by changing the chemical reaction in the melt pool to inhibit the generation of reaction gas. In 2019, Kang et al. [95] promoted the reaction of oxygen and Cr by adding an appropriate amount of Cr particles to the alloy steel powder, thereby inhibiting the carbon–oxygen reaction which could generate gas in the molten pool, thus reducing the porosity of the cladded layer. Li [96] indicated that by baking the powder before LMD, the moisture on the powder’s surface can be dried. This method can not only reduce the porosity caused by the evaporation of water during processing, but also reduce the porosity by inhibiting the chemical reaction of the aluminum alloy powder with water under the laser action. In 2014, Alshaer et al. [97] found that short pulse laser surface cleaning can be used to reduce the porosities of the AC170 PX aluminum-welded coating layers.

Finally, selecting appropriate LMD process parameters, such as laser power, powder feeding rate, scanning speed, etc. [21,23], and remelting processes can also reduce the porosity of the cladded layer. Gao et al. [98] indicated that the remelting process can not only remove pores, but also improve the surface finish of the cladded layer. It is interesting to note that the heat-treatment-related process does not have an large effect on the porosity.

In addition, coupling some physical fields with LMD technology can also reduce the generation of pores. Zhou et al. [99] found that in the LMD process, the application of an electromagnetic force can effectively prevent the appearance of welding pores. In 2018, Zhang et al. [100] combined electric-magnetic compound fields and laser cladding technology, and the results showed that a downward Ampere force can reduce the porosity and the size of the pore. Li et al. [67] indicated that the application of appropriate high-frequency micro-vibrations in laser processing can intensify the flow of the melt materials, thereby promoting the floating of gas and slag in the melt pool, and ultimately achieving the effect of reducing the porosity.

Therefore, corresponding to the reasons for the formation of pores in the LMD process, the methods to reduce the porosity of the processed workpiece mainly include using low porosity powder, inhibiting the chemical reaction of gas generated in the LMD process, choosing the appropriate LMD process parameters or adopting the remelting process method. In addition, coupling some physical fields with LMD can also reduce the generation of pores by affecting the melt flow.

### 4.3. Causes of Crack Formation

In the cladded layer prepared by LMD, the cracks are considered to be the worst defects because they directly cause the workpiece to fail. Crack sensitivity can be described as the crack initiation probability of the cladded layer. For a laser-cladded layer, the cracks are usually perpendicular to the scanning direction [101]. In order to develop methods to suppress the formation of cracks to improve the quality of the cladded layer, it is necessary to explore the crack formation mechanisms.

**Figure 18:**
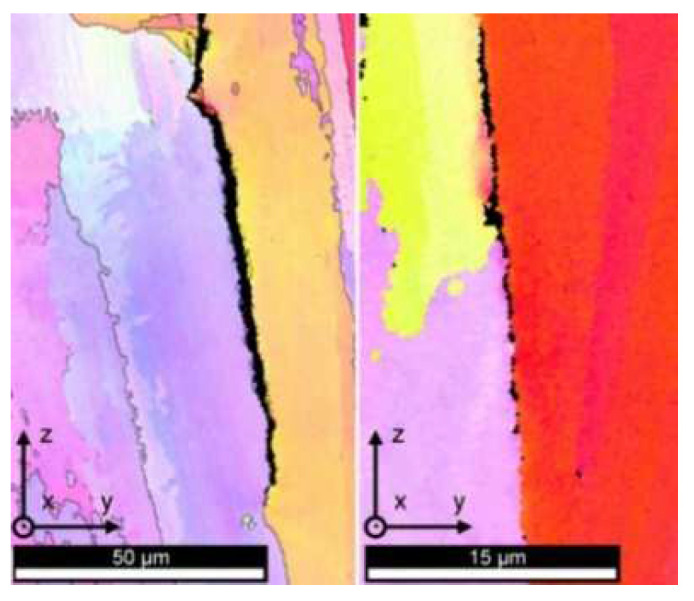
The hot cracks in CZ of the IN 738LC cladded layer (the laser power is 200 w, and the scanning speed is 600 mm/min) [102].

**Figure 19:**
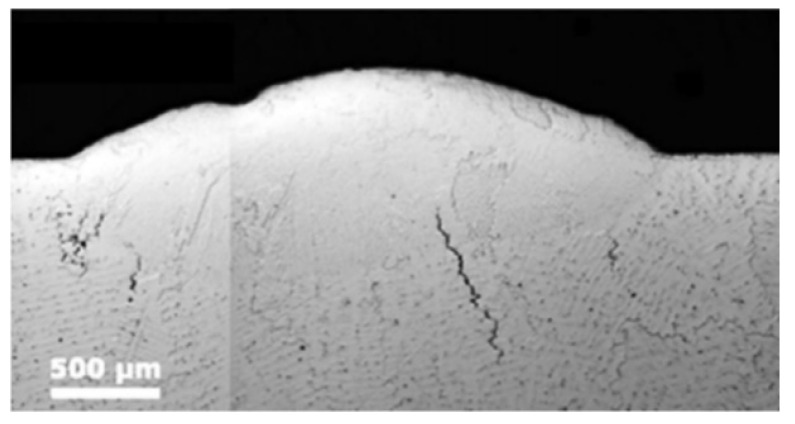
The clod cracks in HAZ of the CM-247LC substrate (the laser power is 1000 w, and the scanning speed is 199 mm/min) [103].

The cracks in the LMD-cladded layer can be divided into hot cracks and cold cracks. The initiation of hot cracks is caused by hot tearing and is affected by the microstructure [101,102], as shown in Figure 18. The cold cracks refer to the cracks caused by the phase transition when the material is heated and cooled to a lower temperature, and also refer to the cracks caused by the excessive thermal strain during melting and solidification due to the different thermal characteristics of the cladding material and the substrate. The cold cracks mainly occur in the heat-affected zone (HAZ) of the cladded layer [103], as shown in Figure 19.

The segregation phenomenon and the uneven distribution of compounds, coarse brittle phases and impurities in the cladded layer are important factors for the formation of hot cracks [101,102,104,105]. In 2018, Wang et al. [101] found that V_2_O_5_ can used as a grain refiner of NiCrBSiC laser-cladded layers. The addition of V_2_O_5_ inhibits the formation of brittle phases and promotes the uniform distribution of most elements and compounds to reduce crack sensitivity. Moreover, the segregation phenomenon in the alloy liquid film between dendrites in the cladded layer is also an important factor leading to cracks. In 2016, Cloots et al. [102] indicated that there is a low-melting, high-concentration Zr liquid film near the crystal grains formed in the cladding zone (CZ), which leads to brittle grain boundaries, and these brittle grain boundaries cannot transmit residual stress and cause cracks. In 2020, Alizadeh-Sh et al. [104] fabricated Inconel 718 laser cladding coating on an A-286 Fe-based superalloy substrate. They found that the dilution rate and impurity elements such as S and P can affect the segregation of dendrites so as to cause cracks. The higher the segregation potential of the alloying elements, the more prone is the substrate to solidification cracks. Nakki et al. [106] found that Inconel 625 with a lower impurity content (such as S, P, B) was the least prone to hot cracking. In addition, the thicker the dendrites are, the more likely the alloy liquid will penetrate between the dendrites, which would lead to an increase in the thickness of the intergranular liquid film, and hence a decrease in the critical thermal stresses [107].

Therefore, the cracks in the LMD-cladded layer can be divided into hot cracks and cold cracks. The initiation of hot cracks is generally caused by hot tearing, which is directly related to the segregation phenomenon and the uneven distribution of compounds, coarse brittle phases and impurities in the cladded layer. However, for the initiation of cold cracks, they are generally caused by the phase transition in the heat-affected zone (HAZ) of the cladded layer.

### 4.4. Methods of Crack Suppression

In order to prepare laser-cladded workpieces with good quality and high reliability, suppressing the formation of cracks in the cladded layer has become the current research frontier of LMD technology. Due to the different causes of cold cracks and hot cracks, the methods for reducing crack sensitivity are also different.

**Figure 20:**
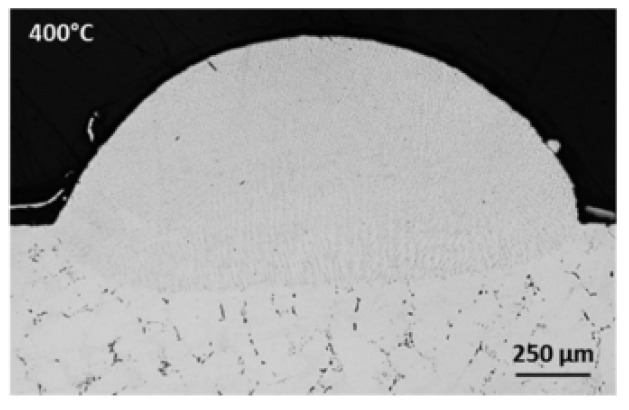
Cracks in HAZ of the CM-247LC substrate disappeared after 400 °C induction preheating (the laser power is 320 w, and the scanning speed is 50 mm/min) [103].

For cold cracks, the method of preheating the substrate can be used to reduce the crack sensitivity of the heat-affected zone (HAZ) in the substrate. In 2020, Bidron et al. [103] proposed that, compared to the substrate without induction preheating (Figure 19), the induction preheating of the substrate can change the precipitated phases in the substrate, thereby reducing the crack sensitivity of the HAZ, as shown in Figure 20. It is worth noting that appropriately reducing the power input or increasing the laser scanning speed can also reduce the crack sensitivity of the heat-affected zone (HAZ) [108].

For hot cracks, the crack sensitivity of the cladded layer can be reduced by refining the grains of the cladded layer. There are three main methods for refining the grains of the cladded layer: controlling the process parameters, coupling the physical fields, and adding rare earth powders.

**Figure 21:**
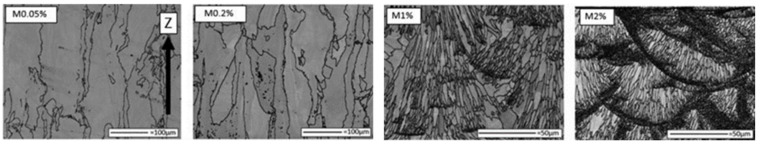
Electron Back-scattering Pattern (EBSD) images exhibit microstructures according to the volumetric quantity of added YSZ [109].

Different alloy powders have different microstructures under the same process parameters. In general, however, when the lower laser power and higher scanning speed are combined, a finer microstructure in the cladded layer is usually formed due to the low input energy and high cooling rate, while a higher laser power and lower scanning speed will increase the incident energy and reduce the cooling rate, resulting in a coarser microstructure in the cladded layer [21,23,62,108]. Furthermore, increasing the powder feeding rate will cause the unmelted powder to become nucleation sites for equiaxed grains and hence refine the microstructure of the cladded layer [22]. Coupling the LMD process with different physical fields, such as high-frequency vibrations [67], ultrasonic vibrations [68,69], electric fields [70], magnetic fields [73,74], etc., can promote the refinement of the crystal grains in the melt pool by breaking dendrites, thereby reducing the crack sensitivity of the cladded layer. Another method for refining the grains of the cladded layer is to add rare earth powder to the processed alloy powder. In 2020, Opprecht et al. [109] added various quantities of Yttrium Stabilized Zirconia (YSZ) to an Al6061 powder to form Al_3_Zr as the nucleation sites for equiaxed grain in a molten state to induce grain refinement and suppress the formation of columnar grains and hence eliminate the hot cracks of the 6061 alloy in laser cladding. The experimental results showed that as the amount of YSZ increases, the equiaxed grain zone extends to the center of the melt pool, as shown in Figure 21. Meanwhile, the length and number of cracks in the cladding zone are significantly reduced or even eliminated. Wang et al. [55] indicated that La, as a surface-active element, is mainly distributed on the grain boundaries, and that the use of La_2_O_3_ is beneficial to refine the grains and microstructure of the Fe-based cladded layer under oil lubrication condition.

In the LMD process, in order to suppress the formation of cold cracks, a method of preheating the substrate can be used, while in order to suppress the formation of hot cracks, the goal is mainly achieved by refining the grains of the cladded layer. The methods of refining the grains of the cladded layer mainly include three methods: changing the process parameters, adding physical fields to the LMD technology and adding rare earth materials in the powder.

It should be noted that the roughness has an important influence on the wear resistance and corrosion resistance of the cladded layer. Chen et al. [110] showed that the cladding speed has a significant effect on the surface quality. Generally, the roughness decreased with the increase in the scanning speed. In addition, a high overlap rate reduced the surface fluctuation of the coating and improved the surface quality of the cladded layer. Therefore, the roughness of the cladded layer can be improved by optimizing the process parameters. In occasions with high requirements or roughness, high-quality cladded surfaces can be obtained by post-machining methods.

To sum up, the most important defects in the cladded layer of LMD technology are pores and cracks. The formation of pores is related to the hollow powder, physical and chemical reactions in the laser cladding process, and cladding process parameters. Improving powder quality and optimizing process parameters can suppress the formation of pore defects. At the same time, external field aids such as electromagnetic fields and ultrasonic vibrations have shown good effects in suppressing pore defects, and will be a hot research direction in the future. The formation of crack defects is closely related to the difference in the thermal expansion characteristics of powder materials and substrate materials, phase distribution and element segregation. Preheating the substrate can reduce the generation of cold cracks, and the use of inoculants such as rare earth powders can refine the grains and significantly reduce the formation of hot cracks; it is considered to be one of the most effective methods to suppress hot crack defects, and has begun to attract more and more researchers’ attention.

## 5. Performance Improvement of Cladding with Different Alloy Powders

Understanding the mechanism of defect formation and developing methods to suppress defects are crucial for the preparation of cladded layers with excellent properties. In order to promote the application of LMD for cladding, it is also necessary to study the performance improvements of cladding with different alloy powders. The LMD technology can process different powder materials on different substrate metal surfaces to prepare cladded layers with an excellent performance. So, it is of great importance to study the performance improvement of cladding from the perspective of powder classification. The cladding powder materials are mainly divided into self-fluxing alloy powders, metal-ceramic composite powders, rare earth alloy powders and functionally gradient materials.

### 5.1. Self-Fluxing Alloy Powder

Self-fluxing alloy powder refers to alloy powder containing Si, B, etc., which has strong deoxidation and slagging capabilities. During laser cladding of self-fluxing alloys, the self-fluxing alloy first chemically reacts with oxygen and oxides in the substrate, borosilicate is then formed on the surface of the molten pool, and the wettability and formability of the substrate and cladded layer has thereby been improved. Self-fluxing alloy powders mainly include Ni-based alloy powder, Fe-based alloy powder, Co-based alloy powder and Cu-based alloy powder. Different alloy powder materials have been applied to different substrates to prepare cladded layers with better performance than that of the substrates. Studying the properties of cladded layers prepared by laser cladding with different self-fluxing alloy powders is the basis for further research on metal–ceramic composite powders and rare earth alloy powders, which has an extremely important significance.

**Figure 22:**
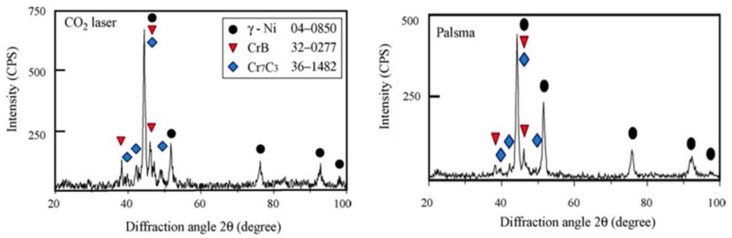
Results of X-ray diffraction analysis of WELPC-6 laser-cladded layers [112].

The cladded layer prepared with Ni-based powder has characteristics of high toughness, good wear resistance and a good processing performance [111]. In 2014, Xu et al. [112] fabricated Ni-based alloy (WELPC-6 alloy) coatings on 316L substrates using a CO_2_ laser and plasma, respectively. The X-ray diffraction of the cladded layers by laser cladding (the laser power is 2 kw, and the scanning speed is 3.85 mm/min) and plasma cladding (processed at 58–68 A and 26 V) are shown in Figure 22. The phase composition of the two cladding processes is the same (Ni-rich solid solution (γ-Ni), borides and carbides such as CrB, Cr_7_C_3_), but the cladding layer fabricated by laser cladding technology shows a fine structure, low dilution, high Vickers hardness and excellent wear resistance. The Ni and Al elements can form intermetallic compounds, which easily form a metallurgy bonding with the aluminum alloy substrate, and can significantly improve the hardness and wear resistance of the cladded layer [113]. In 2015, Wu et al. [114] fabricated Ni-based composite coatings on aluminum alloy by laser cladding technology, and the microstructure shows that due to the appearance of Ni–Al and (Ni, Cr, Fe)_x_C_y_ intermetallic compounds, the microhardness and wear resistance of the cladding layer are significantly improved. In 2017, Chen et al. [115] successfully clad Ni-based alloy on the surface of worn TC_2_ substrate using LMD technology. The experimental results showed that the Ni-based alloy cladded layer processed by laser cladding technology can repair the worn surface of TC_2_ titanium alloy, and the microhardness of the prepared cladded layer is significantly higher than that of the substrate. In 2018, Wang et al. [116] found that after cladding a nickel-based alloy coating on a copper substrate, the microhardness of the cladded layer was increased by more than 4 times and the wear resistance was increased by 14 times compared with that of copper substrate.

**Figure 23:**
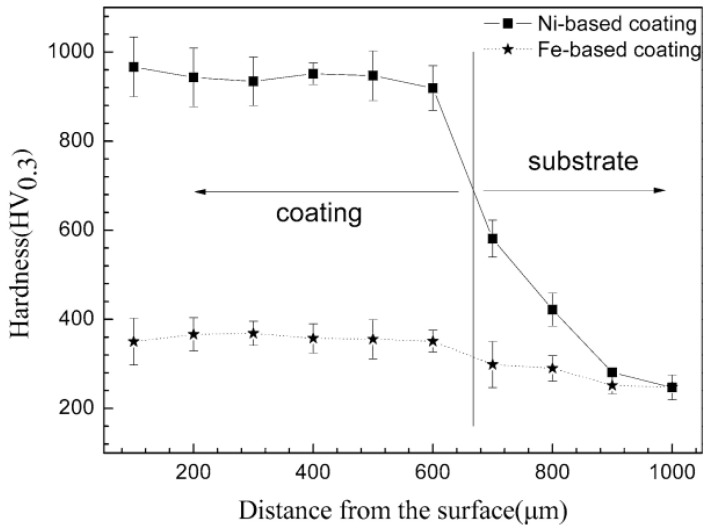
Microhardness of the Ni-based and Fe-based coatings on 40Cr steel (the laser power is 2000 w, and the scanning speed is 90 mm/min) [118].

**Figure 24:**
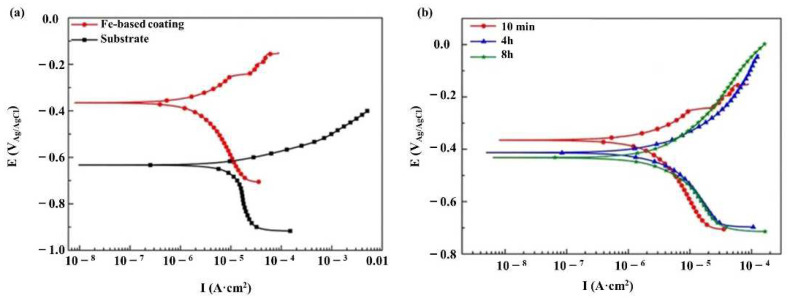
Potentio-dynamic polarization curves of the Fe-based coating with various immersion times in 3.5 wt.% NaCl solution: (**a**) 10 min immersion time; (**b**) 10 min, 4 h, 8 h immersion time [118].

The cladded layer prepared with Fe-based powder has characteristics of a low cost, good mechanical properties, good compatibility with cast iron and low carbon steel substrates, and strong interface bonding. It is one of the most commonly used materials for the preparation of wear-resistant cladded layers [117]. In 2018, Wan et al. [118] deposited Ni-based (5.9 wt%C 3.7 wt%Si, 17.1 wt%Cr 3.6 wt%B, 7.7 wt%Fe, Bal. Ni) and Fe-based (0.03 wt% C, 0.8 wt%Si, 17.3wt%Cr, 2 wt%Ni, 1.15 wt%Mo, Bal. Fe) composite alloy cladded layers on 40Cr steel, respectively, to improve the surface mechanical property of 40Cr steel. The results showed that the Ni-based cladded layer is mainly composed of γ-(Ni, Fe), FeNi_3_, Ni_31_Si_12_, Ni_3_B, CrB and Cr_7_C_3_, and the Fe-based cladded layer is mainly composed of austenite and (Fe, Cr)_7_C_3_. As shown in Figure 23, the microhardness of the Ni-based cladded layer is about 960HV0.3, which is much higher than that of the Fe-based cladded layer (357.4HV0.3) and the 40Cr substrate (251HV0.3). It is also interesting to mention that the electrochemical performance of the coating was evaluated by electrochemical experiments. As seen in the potentio-dynamic polarization curves of Fe-based cladded layers (Figure 24), the corrosion current of the Fe-based cladded layer (11.1 μA/cm^2^) is much lower than that of the substrate (25.75 μA/cm^2^), so that the Fe-based cladded layer fabricated by laser cladding has significant advantages in improve the electro-chemical corrosion resistance of the substrate (40Cr steel). Chen et al. [119] produced Fe-based coating on a pure Ti substrate by laser cladding technology. It can be found from the experiment that the prepared coating is mainly composed of Fe, Fe_2_Ti, Fe_2_B, Fe_3_Si, Ti_2_Ni and Fe_2_O_3_ phases, and the Fe-based coating gives a high average hardness of approximately 860HV0.2, which is about 4.5 times than that of pure Ti substrate (approximately 190HV0.2). In addition, compared with pure Ti substrate, the wear resistance of Fe-based alloy cladded layers has also been significantly improved (the average wear rate of the substrate is 10^−4^ mm^3^/(N·m), while the average wear rate of the cladded layer is (0.70–2.32) × 10^−6^ mm^3^/(N·m). In 2016, Jiang et al. [120] prepared an Fe-based coating on QA19-4 alloys’ surfaces by the CO_2_ laser cladding process. They indicated that the Fe-based coating is mainly composed of γ-(Fe-Ni), CrFe_4_ and Cu_3.8_Ni phases. The microhardness of the cladded layer is at most twice than that of the substrate material, and the wear resistance is 2 times higher than that of the substrate material. Peng et al. [121] prepared cladded layers with the mixed powder of pure Fe powder and Al powder in four different proportion on the surface of ZL114A aluminum alloys using laser cladding technology. The cladded layer is comprised of FeAl, Fe_3_Al, Fe_2_Al_5_ and FeAl_3_ phases. The hardness of the four cladded layers is 3–6 times higher than that of the substrate, and the wear resistance of the cladded layers in different proportions were improved to different degrees in comparison to the substrate.

**Figure 25:**
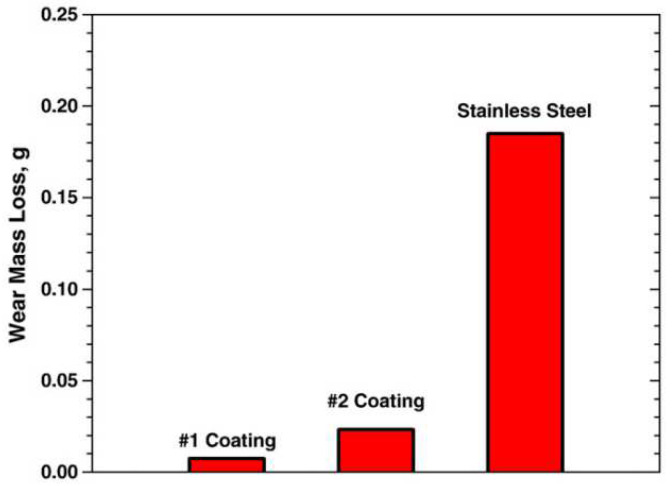
Wear mass loss of the laser-clad Co_3_Mo_2_Si/Co coatings in comparison with the stainless steel [124].

The Co element in the Co-based alloy mainly reacts with Cr, Mo, Si and other elements to form a strengthening phase, which is evenly distributed in the Co-based coating to produce a strengthening effect and improve the coating performance [122]. In 2016, Yang et al. [123] investigated the microstructure and properties of Co-based alloy laser cladding on Ni-based alloys. It was indicated that the microhardness of the Co-based alloy composite cladded layer is about 2.6 times higher than that of the Inconel 600 substrate, and the wear resistance of the cladded layer is 36.6 times higher than that of the Inconel 600 substrate. By using laser cladding technology, Liu et al. [124] prepared wear resistant Co_3_Mo_2_Si/Co coatings on the surface of austenitic stainless steel. As seen in Figure 25, compared with the stainless steel substrate, the wear resistance of Co_3_Mo_2_Si/Co laser-cladded layers is greatly improved, and for the cladded layer with higher volume fractions of Co_3_Mo_2_Si, the wear mass loss is 1/25 that of the substrate. In 2020, Guo et al. [125] indicated that the Co-based alloy cladded layer on the copper alloy surface via laser cladding is of excellent quality. The hardness of the cladded layer is 645.5 HV, which is significantly higher than that of the substrate (125.1 HV), and the wear resistance of the cladded layer is also improved. In addition, the Co-based alloy cladded layer shows good corrosion resistance, mainly because the surface of Co-based alloys easily forms dense oxidation, and hence the film prevents corrosion from proceeding.

Cu-based alloy powder is composed of the Cu element as the matrix along with an appropriate amount of Ni, Cr, B, Si, Mn and other elements. This kind of alloy powder has characteristics of good mechanical properties, high plasticity, good processability, good corrosion resistance and a low friction coefficient, but Cu also has the disadvantage of poor laser absorption [126]. Chi et al. [127] deposited a Cu-based alloy coating on the surface of Al6061 using laser cladding. They found that the coating consists of a (Cu, Ni) solid solution, with Cu_9_Al_4_, AlFe_0.23_Ni_0.77_ and CoFe phases. The surface hardness of the coating is 4.5 times higher than that of 6061 aluminum alloy. The wear volume is about 30% of the aluminum alloy substrate. The friction factor is reduced to 70% of the substrate. Li et al. [128] prepared a aluminum bronze coating on a magnesium surface by laser cladding. It was indicated that the cladded layer consists of AlCu and CuMg_2_ phases. The maximum hardness of the coating is about 370.2HV0.2, increased 6.4 times compared to the substrate, and the mass of the coating is only 50% of the substrate. Zhang et al. [129] prepared Cu–Mo–Si coatings on steel using laser cladding. The phases appearing in the coatings are Cu, MoSi_2_, Mo_5_Si_3_ and Cu_3_Si. Compared with the substrate, the hardness and wear resistance of the coatings are greatly improved.

In summary, the self-fluxing alloy powder mainly includes Ni-based alloy powders, Fe-based alloy powders, Co-based alloy powders and Cu-based alloy powders. For different substrates, the powder materials used in LMD to prepare the cladded layer are also different. Generally, the powder materials used in the cladded layer have properties better than the substrate materials, and the self-fluxing alloy powder material itself or between it and the substrate will generate intermetallic compounds, solid solutions and hard particles under the laser action, thereby further improving the mechanical properties of the cladded layer. Table 2 summarizes the different self-fluxing alloy powders and corresponding substrates applied in the LMD processes.

It should be noted that the processing parameters are critical to the quality of the cladded layers of various alloys. The grain morphology can be improved, and defects such as pores and cracks can be reduced by optimizing the process parameters. An appropriate combination of process parameters can obtain high-quality cladded layers. The above discussion of the cladding quality of self-fluxing alloys is the performance after optimizing the process parameters. Cladded layers with higher performance requirements can be achieved through metal–ceramic composites, rare metal modification and functionally graded material.

### 5.2. Metal–Ceramic Composite Powder

**Figure 26:**
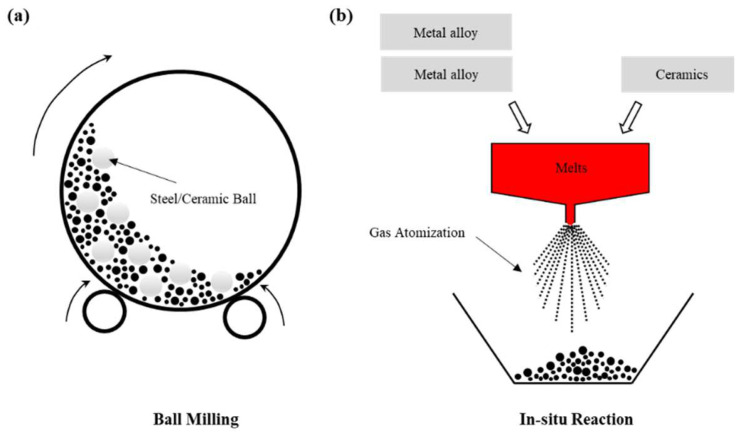
Schematic diagram of ball milling and in-situ reactions: (**a**) ball milling; (**b**) in-situ reactions.

Metal–ceramic composite powder refers to an alloy powder composed by mixing metal materials and ceramic materials or materials which can produce ceramics [122]. Metal–ceramic composites are usually obtained by ball milling and in-situ reactions. Ball milling is the method to obtain metal–ceramic composites by mechanical mixing metal powders and ceramic powders (usually nanoscale) are mixed in a certain proportion and then fully mixed in a ball milling machine. In-situ reactions require the introduction of ceramic particles during the powder manufacturing stage, and a ceramic phase has been produced in the powder obtained by in-situ reactions. The schematic diagram of ball milling and in-situ reactions is shown in Figure 26. The commonly used ceramic reinforcing phases are metal carbides (such as WC, TiC, etc.), metal nitrides (such as TiN, etc.) and metal oxides (such as TiO_2_, Al_2_O_3_, ZrO_2_, etc.), as well as non-metallic borides and silicides (such as SiC, etc.), etc. These ceramic materials have a good wear resistance, corrosion resistance, high-temperature resistance and high-temperature oxidation resistance [59].

**Figure 27:**
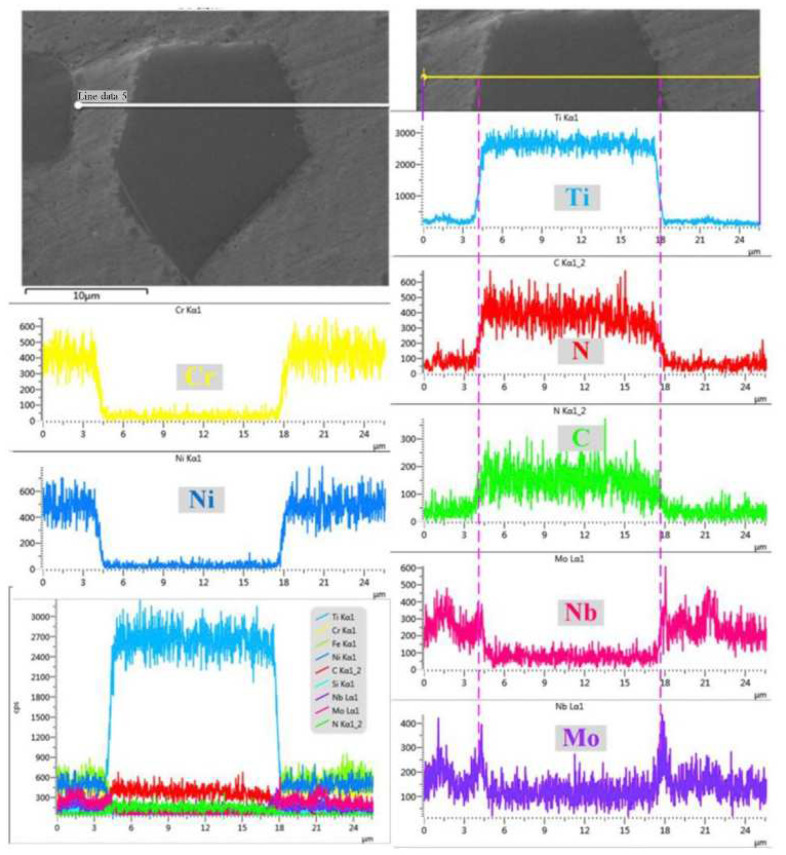
The EDS line scan results of the pentagonal phase (ceramic phase) in Ni204 coating (the laser power is 450 w, and the scanning speed is 330 mm/min) [132].

In 2015, Zhang et al. [130] successfully performed an in-situ cladding of a TiC–VC Fe-based alloy coating on the low = carbon steel substrate. The results showed that the phases of the cladded layer are α-Fe, γ-Fe, TiC, VC and TiVC_2_. The microstructures of the cladded layer matrix are lath martensite and retained austenite. The complex carbides composed of nano TiC, VC and the TiVC_2_ are polygonal blocks uniformly distributed in the cladded layer. The cladded layer with a hardness of 1030HV0.2 possesses good wear and corrosion resistance, which is about 16.85 times than that of the substrate. In 2017, Weng et al. [131] fabricated SiC-reinforced Co-based composite coatings on Ti-6Al-4V titanium alloy substrates, and the results showed that the formation of Ti_5_Si_3_ and TiC can significantly improve the microhardness (the microhardness of the cladded layer is over 3 times that of substrate) and wear resistance (the wear resistance of the cladded layer is over 18.4~57.4 times of that of substrate) of the substrate. In 2017, Zhao et al. [132] fabricated Ni204 coatings with different mass fractions of ceramic particles (TiC, TiN and B_4_C) on the 45-steel substrate. Figure 27 shows the distribution of Ti, N, C, Mo, Nb, Ni, Cr and other elements along the gray-black reinforcement phase (Ti (C, N) ceramic phase) and matrix in the Ni204 coating. The results showed that the addition of these ceramic particles significantly improves the wear resistance of Ni_2_O_4_ composite cladded layers.

Additionally, for the applications involving liquid flows, the cavitation resistance of the cladded later plays an important role. It is generally believed that cavitation resistance is closely related to hardness [133,134,135,136]. Generally, cladding a high-hardness coating on the surface of parts can improve the cavitation resistance. A series of studies have shown that the cavitation resistance can be significantly enhanced by adding ceramic particles such as WC [133,135] and TiC [137], which is closely related to the improvement of the microhardness of the cladding layer.

Therefore, the ceramic phase in the cladded layer can be obtained by ball milling or in-situ reactions [115]. The ceramic-phase-reinforced metal matrix composite cladded layer prepared by LMD on the surface of low-performance substrates not only possesses the strength and toughness of the metal phase material, but also has the high hardness of the ceramic phase material to improve hardness and wear resistance, which has potential applications of huge economic value.

### 5.3. Rare Earth Alloy Powder

The rare earth elements and their oxides have excellent physical and chemical properties due to their special electronic structure and chemical activity. The rare earth elements used for laser cladding to improve the performance of the cladded layer include La, Y, Ce and their oxides.

**Figure 28:**
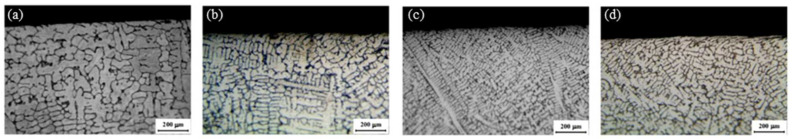
Microstructure of Fe-based alloy laser cladding coating (the laser power is 1.9 kw, and the scanning speed is 200 mm/min): (**a**) without La_2_O_3_; (**b**) with 0.4% La_2_O_3_; (**c**) with 1.2% La_2_O_3_; (**d**) with 2.0% La_2_O_3_ [58].

Wang et al. [58] indicated that the use of La_2_O_3_ is beneficial to refine the microstructure of the Fe-based alloy (0.8–1.2 wt%C, 1.0–2.0 wt%Si 3.8–4.2 wt%B, 16–18 wt%Cr, 9.0–12 wt%Ni, Bal. Fe) cladded layer as shown in Figure 28, and the prepared coating has good wear resistance and contact fatigue damage performance, but it has no significant effect on the surface hardness of the cladded layer. In 2019, Liu et al. [138] fabricated TiC + TiB_2_ reinforced Ni-based composite cladded layers on the surface of Ti811 alloys using LMD. The cladded layer is mainly composed of TiC, TiB_2_, Ti_2_Ni and γ-Ni phases. The Y_2_O_3_ plays a role in grain refinement, dispersion strengthening and increasing the nucleation rate. The cladded layer has high microhardness and good wear resistance. It should be noted that the key in adding rare earth elements to improve the performance of the cladded layer is to control the amount of addition. Too much or too little amount of rare earth cannot play a significant role, and sometimes can even backfire. There are three forms of rare earth in the cladded layer: segregation and solid solution in the crystal lattice, grain boundary, etc.; forming stable, low-melting-point compounds with oxygen, sulfur, silicon, etc.; forming intermetallic compounds with the metal in the cladded layer [59].

The addition of rare earth elements to the alloy powder can inhibit the growth of some grains, increase the nuclei density and affect the melt flow. Therefore, adding an appropriate amount of rare earth powder to the alloy powder can refine the grain and microstructure of the cladded layer, reduce the crack sensitivity of the cladded layer, and finally, significantly improve the wear resistance of the cladded layer.

### 5.4. Functionally Gradient Material

When the chemical and physical properties of the powder material of the substrate material and the cladded layer are too different, the interface combination between the cladded layer and the substrate material will be poor. As a result, the cladded layer could crack or peel off. Therefore, the study of functionally gradient materials to overcome the above problems has become a current research hotspot [121].

**Figure 29:**
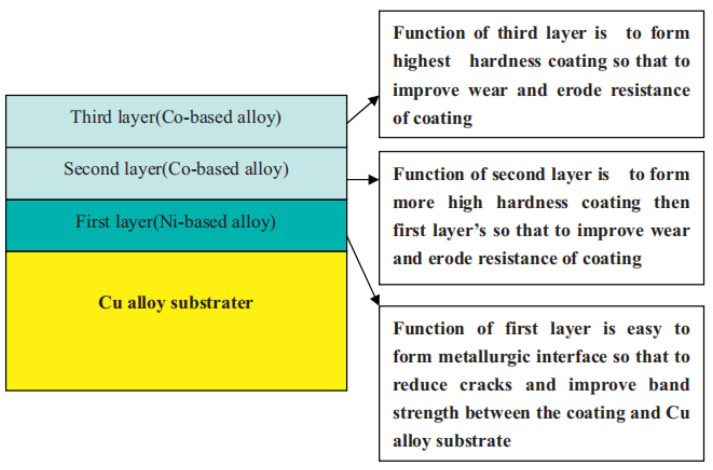
The structure and function of designing three-layer gradient coatings [140].

In 2011, Chen et al. [139] fabricated a high-wear-resistant Ni/Co-based alloy gradient coating on a Cu alloy substrate by laser cladding. As shown in Figure 29, The Cu–Ni–Co high-wear-resistant gradient coating is formed by using a nickel-based alloy as the transition layer. The results showed that the microhardness has been significantly improved (from 98 HV for the Cu substrate to 875 HV for the cladded layer), and the wear resistance of the cladding layer is also increased by 5 times compared with that of the Cu substrate. In 2020, Wang et al. [140] fabricated a laser-cladded gradient coating on a 40Cr gear steel surface using a mixed powder of nano–TiC powder and 12CrNi_2_ powder. They found that with the increase in TiC content, the microhardness of the gradient coating also increased greatly (from 621 HV at the bottom to 1088 HV at the top) because a large amount of TiC agglomerated into a hard phase structure. With the increase in microhardness and the transformation of TiC into a hard phase, the coefficient of friction of the gradient coating is reduced by 50%, and the grinding loss is reduced by 40%. In 2020, Su et al. [141] fabricated Inconel steel functionally graded materials (FGMs), with the graded coating transitioning from 100% 316L incrementally graded (5%, 10%, 20%) to 100% Inconel718. The results showed that the graded coating with a 5% composition gradient has the widest microhardness range (173~308 HV), and the graded coating with a 10% composition gradient has the highest tensile strength (527.05 MPa) and the highest elongation (26.21%).

Functionally graded material coating is a new type of composite material coating whose performance changes with the change in the material gradient. It could be prepared by adjusting a variety of different material compositions according to the required performance and conditions. The functionally graded material coating can solve the problem of coating cracking or peeling caused by the large difference between the physical and chemical properties of the coating material and the substrate material.

Various additives and their enhancement effects are shown in Table 3. In general, the alloy powder material selected for the cladded layer has better mechanical properties, such as hardness, wear resistance and electrochemical corrosion resistance, than the substrate material. The intermetallic compounds, solid solutions and hard particles will be generated in the cladded layer during laser processing to further improve the mechanical properties of the cladded layer. The cladding powder materials are mainly divided into self-fluxing alloy powder, metal–ceramic composite powder, rare earth alloy powder and functionally gradient material. The self-fluxing alloy materials are mainly Ni-based, Fe-based, Co-based and Cu-based alloy materials, which have good mechanical properties and good applicability to the matrix. The structure of the metal–ceramic composite cladded layer contains ceramic phases, which can improve the hardness and wear resistance of the cladded layer. The ceramic phases can be obtained by in-situ synthesis or mechanical addition. The addition of an appropriate amount of rare earth element materials into the cladding alloy powder material can refine the structure, reduce the crack sensitivity and significantly improve the wear resistance of the cladded layer. The functionally graded material coating is a new type of composite material coating whose performance changes with the change in the material gradient. The functionally graded material can solve the problem of coating cracking or peeling caused by the large difference between the physical and chemical properties of the coating material and the substrate material. The functionally gradient material has a very broad development space and application prospects, and it is one of the key directions of future cladding materials research [59,122,142,143,144]. In the LMD process, having a good command of the performance improvement of cladding with different alloy powders is a prerequisite for further popularizing the technology of LMD on the basis of understanding the formation mechanism of cladded layer defects and adopting defect suppression methods to ensure the quality of the cladded layer, which has extremely important practical engineering significance.

## 6. Summary and Outlook

This work reviews previous efforts on the simulation technology of the melt pool grain growth mechanism, temperature and stress distribution that are directly related to defect formation in LMD technology. At the same time, the defect suppression method and the performance improvement method of the cladded layer in the LMD technology are introduced.

In terms of the mechanism of crystal grain growth in the melt pool in the LMD process, the crystal grains in the melt pool are dominated by equiaxed grains on the surface of the melt pool and columnar grains at the bottom. The surface-active elements can affect the depth and shape of the melt pool by influencing the direction of the melt flow driven by the Marangoni double-ring vortex. The flowing of the melt materials can increase the density of crystal nuclei by breaking dendrites to promote the formation of equiaxed grains. It can also affect the growth of columnar grains at the bottom of the melt pool by changing the direction of heat flow. The direction of heat flow in the melt pool is also related to some factors such as the laser scanning strategy and laser incident angle. When the multi-layer cladding is performed, due to the partial remelting of the previous layers, the longer columnar grains in the cladded layer will grow. Based on the mechanism that the formation and growth of crystal grains in the melt pool are affected by the melt flow, adding high-frequency micro-vibrations, ultrasonic vibrations, electric fields, magnetic fields and other physical fields to the LMD technology can be proposed to break the dendrites in the melt pool by affecting the melt flow and hence realize the control of the microstructure of the cladded layer.

At present, the relevant research on the effect of surface-active elements such as S, La, Ce on the melt flow trend in the melt pool during the laser cladding process is still limited. For further quantification and modeling of the relationship between the addition of La, Ce and other surface-active elements and the melt flow trend or the depth of the melt pool, the issues of exploring new surface-active elements and controlling the interior of the melt pool by adjusting the surface-active elements of the melt pool melt flow so as to improve the performance of the cladded layer are the future research directions that need to be further explored.

In the previous work on the evolution and distribution of the temperature and stress, in addition to the obvious temperature gradient near the heat source, the difference between the thermal and physical properties of the cladding powder material and the substrate material will also cause the temperature gradient to be generated, which affects the generation and distribution of thermal stress. Under the influence of the temperature gradient, different phase changes occur in different areas of the cladded layer, resulting in tensile or compressive stress. The thermal stress and tensile or compressive stress together constitutes residual stress. The relationship between stress and strain is affected by the structure of the substrate. Along the direction that the substrate deformation resistance is the greatest, the strain is the smallest, while the residual stress is the largest, and the cracks are most likely to occur. There are several ways to reduce the residual stress of the cladded layer: selecting powder material with a thermal expansion coefficient similar to that of the substrate, selecting a suitable LMD scanning strategy, preheating the substrate and adjusting the processing parameters to reduce the laser energy density.

The main defects of the cladded layer processed by LMD are pores and cracks. The direct causes of the formation of pores are mainly due to the fact that the powder contains pores, the chemical reaction generating gas during the laser processing process and the improper selection of processing parameters. Thus, it is possible to reduce the porosity of the cladded layer by using low-porosity powders, suppressing the chemical reaction of the gas generated in the LMD process, and selecting appropriate LMD processing parameters or adopting a remelting process method. Moreover, coupling some physical fields with LMD technology can also reduce the generation of pores. The cracks are classified into cold cracks and hot cracks. The formation of cold cracks is related to two factors, which are the phase change caused by the heating and cooling of the material and the excessive thermal strain caused by the difference between the thermal characteristics of the powder material and the substrate. The formation of hot cracks is affected by the microstructure of the cladded layer. Thus, the method of preheating the substrate can be used to suppress cold cracks, while the method of grain refinement can be used to suppress the formation of hot cracks. The methods of refining the grains of the cladded layer mainly include three methods: changing the processing parameters, adding physical fields to the LMD technology and adding rare earth materials to the powder.

The processing parameters play an extremely important role in the technology of LMD for cladding. They are closely related to the size of the residual stress of the cladded layer, the shape of the microstructure and the formation of pores and cracks. However, for different metal powders, the optimal processing parameters are usually determined based on the repeated experiments. Therefore, the establishment of a mathematical model with parameters such as the melting point of metal powder, the melting point of the base material and the mass fraction of various materials as independent variables, and related processing parameters as dependent variables, is one of the future research hotspots.

The cladding powder materials are mainly divided into self-fluxing alloy powders, metal–ceramic composite powders, rare earth alloy powders and functionally gradient materials. The self-fluxing alloy materials mainly include Ni-based, Fe-based, Co-based and Cu-based alloy materials. The structure of the metal–cermet composite cladded layer contains ceramic phases, which can improve the hardness and wear resistance of the cladded layer. The ceramic phases can be obtained by in-situ synthesis or mechanical addition. The addition of an appropriate amount of rare earth element materials into the cladding alloy powder material can refine the structure and reduce the crack sensitivity. It can also significantly improve the wear resistance of the cladded layer. The functionally graded material coating is a new type of composite material coating whose performance changes with the change in the material gradient, which is prepared by adjusting a variety of different material compositions according to the required performance and conditions. The functionally graded material coating can solve the problem of coating cracking or peeling caused by the large difference between the physical and chemical properties of the coating material and the substrate material.

Manufacturing functionally graded material workpieces by LMD is an effective way to solve the disadvantages of prepared traditional metal materials such as a low specific strength, low specific rigidity and poor corrosion resistance. The multiphase materials and multiple materials are two ways to achieve functionally graded materials. Therefore, designing and controlling the distribution of metal phases or material components corresponding to different layers of the cladded layer to meet the needs of different working conditions is a hot research topic in the future. On the other hand, the realization of “material–processing–microstructure–performance” is a research hotspot in the future. The traditional manufacturing idea is to design a process based on the selection of materials to form a microstructure and, finally, to obtain a workpiece with certain properties. In the future, the idea of LMD technology is to actively select materials based on the required performance, then combine them with a controllable processing process to form the corresponding microstructure, and finally, to actively realize the expected function.

## Figures and Tables

**Table 1:** The coupling of physical fields and its effects.

Coupling Physical Fields	Substrate/Powder	Effects
High-frequency microvibration [67]	5025Al/AlSi10Mg&TiC	The grain size has been refined
Ultrasonic vibration [68]	Low carbon steel/AISI 630	Acoustic streaming and cavitation effects transform the microstructure into equiaxed grains.
Pulse current [70]	GH4169/FG4169	Promote grain refinement
Strong magnetic field [73]	DZ417G et al.	Fragmentation of cells/dendrites and columnar-to-equiaxed transition
Magnetic field [74]	Fe-based alloy/316L	The equiaxed grain area has been significantly enlarged.

**Table 2:** Different self-fluxing alloy powders and corresponding substrates.

Alloy Powder	Substrate	References
Ni-based	Steel, Al alloy, Ti alloy, Cu alloy	[112,113,114,115,116]
Fe-based	Steel, Al alloy, Ti alloy, Cu alloy	[118,119,120,121]
Co-based	Steel, Ni alloy, Cu alloy	[123,124,125]
Cu-based	Steel, Al alloy, Mg alloy	[127,128,129]

**Table 3:** Various additives and their enhancement effects.

Additives	Powder Materials	Effects
TiC-VC [130]	Fe-based alloy	Wear and corrosion resistance increased
SiC [131]	Co-based alloy	Microhardness increased by more than 3 times, and the wear resistance is increased by 18.4–57.4 times
TiC, TiN and B_4_C [132]	Ni_2_O_4_	Wear resistance increased
La_2_O_3_ [58]	Fe-based alloy	Microstructure refined and the wear resistance improved
TiC + TiB_2_ [138]	Ni-based alloy	Wear resistance and microhardness increased

## Data Availability

Not applicable.

## References

[1] Sexton L., Lavin S., Byrne G., Kennedy A. (2002). Laser Cladding of Aerospace Materials. J. Mater. Process. Technol..

[2] Kattire P., Paul S., Singh R., Yan W. (2015). Experimental Characterization of Laser Cladding of CPM 9V On H13 Tool Steel for Die Repair Applications. J. Manuf. Process..

[3] Song J., Deng Q., Chen C., Hu D., Li Y. (2006). Rebuilding of Metal Components with Laser Cladding Forming. Appl. Surf. Sci..

[4] Farahmand P., Kovacevic R. (2014). An Experimental–Numerical Investigation of Heat Distribution and Stress Field in Single- and Multi-Track Laser Cladding by a High-Power Direct Diode Laser. Opt. Laser Technol..

[5] Schubert E., Seefeld T., Rinn A., Sepold G. (1999). Laser Beam Cladding: A Flexible Tool for Local Surface Treatment and Repair. J. Therm. Spray Technol..

[6] Thompson S.M., Bian L., Shamsaei N., Yadollahi A. (2015). An Overview of Direct Laser Deposition for Additive Manufacturing; Part I: Transport Phenomena, Modeling and Diagnostics. Addit. Manuf..

[7] Brown C.O., Breinan E.M., Kear B.H. (1982). Method for Fabricating Articles by Sequential Layer Deposition. U.S. Patent.

[8] Lewis G.K., Nemec R., Milewski J., Thoma D.J., Cremers D., Barbe M. Directed Light Fabrication. Proceedings of the International Congress on Applications of Lasers & Electro-Optics.

[9] Jeantette F.P., Keicher D.M., Romero J.A., Schanwald L.P. (2000). Method and System for Producing Complex-Shape Objects. U.S. Patent.

[10] Hammeke A.W. (1988). Laser Spray Nozzle and Method. U.S. Patent.

[11] Buongiorno A. (1995). Laser/Powdered Metal Cladding Nozzle. U.S. Patent.

[12] Tan H., Zhang F., Wen R., Chen J., Huang W. (2012). Experiment Study of Powder Flow Feed Behavior of Laser Solid Forming. Opt. Lasers Eng..

[13] Gu D., Meiners W., Wissenbach K., Poprawe P. (2012). Laser additive manufacturing of metallic components: Materials, processes and mechanisms. Int. Mater. Rev..

[14] Saboori A., Gallo D., Biamino S., Fino P., Lombardi M. (2017). An Overview of Additive Manufacturing of Titanium Components by Directed Energy Deposition: Microstructure and Mechanical Properties. Appl. Sci..

[15] Liu D., Lippold J.C., Li J., Rohklin S.R., Vollbrecht J., Grylls R. (2014). Laser Engineered Net Shape (LENS) Technology for the Repair of Ni-Base Superalloy Turbine Components. Metall. Mater. Trans. A.

[16] Zhao X., Chen J., Lin X., Huang W. (2008). Study on Microstructure and Mechanical Properties of Laser Rapid Forming Inconel 718. Mater. Sci. Eng. A.

[17] Sun X., Zhang J., Pan W., Wang W., Tang C. (2022). Research progress in surface strengthening technology of carbide-based coating. J. Alloys Compd..

[18] Shamsaei N., Yadollahi A., Bian L., Thompson S.M. (2015). An Overview of Direct Laser Deposition for Additive Manufacturing; Part II: Mechanical Behavior, Process Parameter Optimization and Control. Addit. Manuf..

[19] Wang D., Tian Z., Shen L. (2009). Microstructural characteristics and formation mechanism of Al_2_O_3_-13wt.%TiO_2_ coatings plasma-sprayed with nanostructured agglomerated powders. Surf. Coat. Technol..

[20] Ju H., Lin C., Liu Z., Zhang J. (2018). Study of in-situ formation of Fe-Mn-Si shape memory alloy welding seam by laser welding with filler powder. Opt. Laser Technol..

[21] Kumar P., Farah J., Akram J., Teng C., Ginn J., Misra M. (2019). Influence of Laser Processing Parameters on Porosity in Inconel 718 During Additive Manufacturing. Int. J. Adv. Manuf. Technol..

[22] Wang T., Zhu Y.Y., Zhang S.Q., Tang H.B., Wang H.M. (2015). Grain Morphology Evolution Behavior of Titanium Alloy Components During Laser Melting Deposition Additive Manufacturing. J. Alloys Compd..

[23] Ng G.K.L., Jarfors A.E.W., Bi G., Zheng H.Y. (2009). Porosity Formation and Gas Bubble Retention in Laser Metal Deposition. Appl. Phys. A.

[24] Chen J., Tian D., Zhan X., Qi C., Zhou J. (2019). Study on Grain Size of Deposition Layer in Single-Layer Laser Melting Deposition Additive Manufacturing on Invar Alloy. Opt. Eng..

[25] Tian M., Shi S., Fu G. (2013). Study on Laser Rapid Prototyping of High Thin-walled Revolved Parts with Variable Diameters based on Inside-laser Coaxial Powder Feeding. Appl. Mech. Mater..

[26] Weng F., Yu H., Chen C., Liu J., Zhao L., Dai J., Zhao Z. (2017). Effect of Process Parameters on the Microstructure Evolution and Wear Property of the Laser Cladding Coatings on Ti-6Al-4V Alloy. J. Alloys Compd..

[27] Zhu Y., Liu D., Tian X., Tang H., Wang H. (2014). Characterization of Microstructure and Mechanical Properties of Laser Melting Deposited Ti-6.5Al-3.5Mo-1.5Zr-0.3Si Titanium Alloy. Mater. Des..

[28] Zhang F., Qiu Y., Hu T., Clare A.T., Li Y., Zhang L.-C. (2020). Microstructures and Mechanical Behavior of Beta-Type Ti-25V-15Cr-0.2Si Titanium Alloy Coating by Laser Cladding. Mater. Sci. Eng. A.

[29] Song W., Echigoya J., Zhu B., Xie C., Huang W., Cui K. (2000). Vacuum Laser Cladding and Effect of Hf on the Cracking Susceptibility and the Microstructure of Fe-Cr-Ni Laser-Clad Layer. Surf. Coat. Technol..

[30] Zhang D., Kao X., Li J. (2016). Research on the process of laser cladding iron-based alloy powder on H13 steel surface. Mach. Des. Manuf..

[31] Chen J., Li X., Xue Y. (2019). Friction and Wear Properties of Laser Cladding Fe901 Alloy Coating On 45 Steel Surface. Chin. J. Lasers.

[32] Shang F., Chen S., Zhang C., Liang J., Liu C., Wang M. (2021). The Effect of Si and B on Formability and Wear Resistance of Preset-Powder Laser Cladding W10V5Co4 Alloy Steel Coating. Opt. Laser Technol..

[33] Zhang Y., Tu Y., Xi M., Shi L. (2008). Characterization on Laser Clad Nickel Based Alloy Coating on Pure Copper. Surf. Coat. Technol..

[34] Lei J., Shi C., Zhou S., Gu Z. (2018). Enhanced Corrosion and Wear Resistance Properties of Carbon Fiber Reinforced Ni-based Composite Coating by Laser Cladding. Surf. Coat. Technol..

[35] Zhang T., Li P., Zhou J., Wang C., Meng X., Huang S. (2021). Microstructure Evolution of Laser Cladding Inconel 718 Assisted Hybrid Ultrasonic-Electromagnetic Field. Mater. Lett..

[36] Hao J., Hu F., Le X., Liu H., Yang H., Han J. (2021). Microstructure and High-Temperature Wear Behaviour of Inconel 625 Multi-Layer Cladding Prepared on H13 Mould Steel by a Hybrid Additive Manufacturing Method. J. Mater. Process. Technol..

[37] Bhowmik A., Yang Y., Zhou W., Chew Y., Bi G. (2021). On the Heterogeneous Cooling Rates in Laser-Clad Al-50Si Alloy. Surf. Coat. Technol..

[38] Wang T., Dai S., Liao H., Zhu H. (2020). Pores and the Formation Mechanisms of SLMed AlSi10Mg. Rapid Prototyp. J..

[39] Chen T., Liu X., Zhao L., Hu L., Li J., Qu F., Le G., Qi L., Wang X. (2020). Investigation on the Two-Stage Hierarchical Phase Separation in the Laser Cladded Cu-Mn-Fe Coating. Vacuum.

[40] Adak B., Nash P., Chen D. (2005). Microstructural Characterization of Laser Cladding of Cu-30Ni. J. Mater. Sci..

[41] Wu Q., Zheng H., Xu Y., Fan C., Zhong N. (2020). Wear Behavior of a Single-Phase Laser-Clad Cu-9Ni-6Sn Coating in Air and in NaCl Solution. J. Mater. Eng. Perform..

[42] Paul C.P., Alemohammad H., Toyserkani E., Khajepour A., Corbin S. (2007). Cladding of WC-12Co On Low Carbon Steel Using a Pulsed Nd:YAG Laser. Mater. Sci. Eng. A.

[43] Liu F., Liu C., Chen S., Tao X., Zhang Y. (2010). Laser Cladding Ni-Co Duplex Coating on Copper Substrate. Opt. Laser Technol..

[44] Qi H., Azer M., Singh P. (2010). Adaptive Toolpath Deposition Method for Laser Net Shape Manufacturing and Repair of Turbine Compressor Airfoils. Int. J. Adv. Manuf. Technol..

[45] Liu Q., Janardhana M., Hinton B., Brandt M., Sharp K. (2011). Laser Cladding as a Potential Repair Technology for Damaged Aircraft Components. Int. J. Struct. Integr..

[46] Lin C. (2015). Parameter Optimization of Laser Cladding Process and Resulting Microstructure for the Repair of Tenon on Steam Turbine Blade. Vacuum.

[47] Paydas H., Mertens A., Carrus R., Lecomte-Beckers J., Tchuindjang J.T. (2015). Laser Cladding as Repair Technology for Ti-6Al-4V Alloy: Influence of Building Strategy on Microstructure and Hardness. Mater. Des..

[48] Torims T., Pikurs G., Ratkus A., Logins A., Vilcans J., Sklariks S. (2015). Development of Technological Equipment to Laboratory Test In-Situ Laser Cladding for Marine Engine Crankshaft Renovation. Procedia Eng..

[49] Liu Q., Wang Y., Zheng H., Tang K., Li H., Gong S. (2016). TC17 Titanium Alloy Laser Melting Deposition Repair Process and Properties. Opt. Laser Technol..

[50] Liu H., Hu Z., Qin X., Wang Y., Zhang J., Huang S. (2017). Parameter Optimization and Experimental Study of the Sprocket Repairing Using Laser Cladding. Int. J. Adv. Manuf. Technol..

[51] Li J. (2014). Research on the geometrical feature and the molten pool’s surface tension of laser cladding layer. Master’s Thesis.

[52] Kumar A., Roy S. (2009). Effect of Three-Dimensional Melt Pool Convection on Process Characteristics During Laser Cladding. Comput. Mater. Sci..

[53] Heiple C.R., Roper J.R. (1982). Mechanism for Minor Element Effect on GTA Fusion Zone Geometry. Weld. J..

[54] Gan Z., Yu G., He X., Li S. (2017). Surface-Active Element Transport and its Effect on Liquid Metal Flow in Laser-Assisted Additive Manufacturing. Int. Commun. Heat Mass Transf..

[55] Hu Y., Wang G., Ye M., Wang S., Wang L., Rong Y. (2018). A precipitation hardening model for Al-Cu-Cd alloys. Mater. Des..

[56] Aucott L., Dong H., Mirihanage W., Atwood R., Kidess A., Gao S., Wen S., Marsden J., Feng S., Tong M. (2018). Revealing Internal Flow Behaviour in Arc Welding and Additive Manufacturing of Metals. Nat. Commun..

[57] Sun S., Fu H., Ping X., Guo X., Lin J., Lei Y., Wu W., Zhou J. (2019). Effect of CeO_2_ Addition On Microstructure and Mechanical Properties of In-Situ (Ti, Nb)C/Ni Coating. Surf. Coat. Technol..

[58] Wang W.J., Fu Z.K., Cao X., Guo J., Liu Q.Y., Zhu M.H. (2016). The Role of Lanthanum Oxide on Wear and Contact Fatigue Damage Resistance of Laser Cladding Fe-based Alloy Coating Under Oil Lubrication Condition. Tribol. Int..

[59] Zhang X., Liu H., Jiang Y., Wang C. (2012). Research Progress of Functional Composite Coatings on Ti6Al4V Alloy Surface Prepared by Laser Cladding Technique. Rare Met. Mater. Eng..

[60] Gäumann M., Henry S., Cléton F., Wagnière J.-D., Kurz W. (1999). Epitaxial Laser Metal Forming: Analysis of Microstructure Formation. Mater. Sci. Eng. A.

[61] Zhang Y., Yang L., Dai J., Huang Z., Meng T. (2016). Grain Growth of Ni-based Superalloy IN718 Coating Fabricated by Pulsed Laser Deposition. Opt. Laser Technol..

[62] Thijs L., Verhaeghe F., Craeghs T., Van Humbeeck J., Kruth J.P. (2010). A Study of the Microstructural Evolution During Selective Laser Melting of Ti–6Al–4V. Acta Mater..

[63] Yu J., Lin X., Ma L., Wang J., Fu X., Chen J., Huang W. (2011). Influence of Laser Deposition Patterns on Part Distortion, Interior Quality and Mechanical Properties by Laser Solid Forming (LSF). Mater. Sci. Eng. A.

[64] Gäumann M., Bezencon C., Canalis P., Kurz W. (2001). Single-Crystal Laser Deposition of Superalloys: Processing-Microstructure Maps. Acta Materlalia.

[65] Wang G., Liang J., Zhou Y., Zhao L., Jin T., Sun X. (2018). Variation of Crystal Orientation During Epitaxial Growth of Dendrites by Laser Deposition. J. Mater. Sci. Technol..

[66] Chen Y., Lu F., Zhang K., Nie P., Hosseini S.R.E., Feng K., Li Z., Chu P. (2016). Investigation of Dendritic Growth and Liquation Cracking in Laser Melting Deposited Inconel 718 at Different Laser Input Angles. Mater. Des..

[67] Li C., Sun S., Liu C., Lu Q., Ma P., Wang Y. (2019). Microstructure and Mechanical Properties of TiC/AlSi10Mg Alloy Fabricated by Laser Additive Manufacturing Under High-Frequency Micro-Vibration. J. Alloys Compd..

[68] Cong W., Ning F. (2017). A Fundamental Investigation on Ultrasonic Vibration-Assisted Laser Engineered Net Shaping of Stainless Steel. Int. J. Mach. Tools Manuf..

[69] Zhang S., Zhao Y., Cheng X., Chen G., Dai Q. (2009). High-Energy Ultrasonic Field Effects on the Microstructure and Mechanical Behaviors of A356 Alloy. J. Alloys Compd..

[70] Xie D., Zhao J., Qi Y., Li Y., Shen L., Xiao M. (2013). Decreasing pores in a laser cladded layers with pulsed current. Chin. Opt. Lett..

[71] Barnak J.P., Sprecher A.F., Conrad H. (1995). Colony (Grain) Size Reduction in Eutectic TiC Pb-Sn Castings by Electroplusing. Scr. Matallurgica Mater..

[72] Nakada M., Shiohara Y., Flemings M.C. (1990). Modification of Solidification Structures by Pulse Electric Discharging. ISIJ Int..

[73] Li X., Gagnoud A., Fautrelle Y., Ren Z., Moreau R., Zhang Y., Esling C. (2012). Dendrite Fragmentation and Columnar-To-Equiaxed Transition During Directional Solidification at Lower Growth Speed Under a Strong Magnetic Field. Acta Materlalia.

[74] Zhao D., Xu L., Fu Y. (2021). Effects of molecule force on free vibration for a micro electromagnetic harmonic drive system. Mech. Ind..

[75] Fu Y., Qi T., Zong L. (2017). Effects of Alternating Magnetic Field on Microstructure and Machanics Properties of High Hardness Cladding Layers. China Mech. Eng..

[76] Zhang Y., Li Z., Nie P., Wu Y. (2013). Effect of ultrarapid cooling on microstructure of laser cladding IN718 coating. Surf. Eng..

[77] Lisiecki A., Slizak D., Kukofka A. (2019). Laser cladding of co-based metallic powder at cryogenic conditions. J. Achiev. Mater. Manuf. Eng..

[78] Lisiecki A., Slizak D. (2020). Hybrid laser deposition of fe-based metallic powder under cryogenic conditions. Metals.

[79] Zhou J., Qiu C.J., Cheng X.Y. (2011). Finite Finite Element Simulation of Temperature Field on Laser Cladded layers Regulated by Micro-forging. Adv. Mater. Res..

[80] Anusha E., Kumar A., Shariff S. (2020). A novel method of laser surface hardening treatment inducing different thermal processing condition for Thin-sectioned 100Cr6 steel. Opt. Laser Technol..

[81] Zhang Q., Xu P., Zha G., Ouyang Z., He D. (2021). Numerical simulations of temperature and stress field of Fe-Mn-Si-Cr-Ni shape memory alloy coating synthesized by laser cladding. Optik.

[82] Liu H., Qin X., Wu M., Ni M., Huang S. (2019). Numerical Simulation of Thermal and Stress Field of Single-Track Cladding in Wide-Beam Laser Cladding. Int. J. Adv. Manuf. Technol..

[83] Zheng L., Xie W., Li Y. (2011). The Numerical Simulation on the Temperature Field of Laser Cladding. Key Eng. Mater..

[84] Dai D.P., Jiang X.H., Cai J.P., Lu F., Chen Y., Li Z.G., Deng D. (2015). Numerical Simulation of Temperature Field and Stress Distribution in Inconel718 Ni Base Alloy Induced by Laser Cladding. Chin. J. Laser.

[85] Dai K., Shaw L. (2002). Distortion Minimization of Laser-Processed Components through Control of Laser Scanning Patterns. Rapid Prototyp. J..

[86] Yang X., Zeng R., Fu X., Wang X., Zhou J., Yu L. (2022). Influence of the Cu content on the electrochemical corrosion performances of Ni60 coating. Corros. Sci..

[87] Choo H., Sham K.-L., Bohling J., Ngo A., Xiao X., Ren Y., Depond P.J., Matthews M.J., Garlea E. (2019). Effect of Laser Power on Defect, Texture, and Microstructure of a Laser Powder Bed Fusion Processed 316L Stainless Steel. Mater. Des..

[88] Sun Z., Tan X., Tor S.B., Yeong W.Y. (2016). Selective Laser Melting of Stainless Steel 316L with Low Porosity and High Build Rates. Mater. Des..

[89] Cunningham R., Narra S.P., Ozturk T., Beuth J., Rollett A.D. (2016). Evaluating the Effect of Processing Parameters on Porosity in Electron Beam Melted Ti-6Al-4V Via Synchrotron X-ray Microtomography. J. Miner. Met. Mater. Soc..

[90] Zhang C., Bao Y., Zhu H., Nie X., Zhang W., Zhang S., Zeng X. (2019). A Comparison Between Laser and TIG Welding of Selective Laser Melted AlSi10Mg. Opt. Laser Technol..

[91] Tang M., Chris Pistorius P. (2017). Oxides, Porosity and Fatigue Performance of AlSi10Mg Parts Produced by Selective Laser Melting. Int. J. Fatigue.

[92] Liao H., Zhu H., Xue G., Zeng X. (2019). Alumina Loss Mechanism of Al2O3-AlSi10 Mg Composites During Selective Laser Melting. J. Alloys Compd..

[93] Fu Y., Loredo A., Martin B., Vannes A.B. (2002). A Theoretical Model for Laser and Powder Particles Interaction During Laser Cladding. J. Mater. Processing Technol..

[94] Langebeck A., Bohlen A., Freisse H., Vollertsen F. (2020). Additive Manufacturing with the Lightweight Material Aluminium Alloy EN AW-7075. Weld. World.

[95] Kang H., Dong Z., Zhang W., Xie Y., Peng X. (2019). Laser Melting Deposition of a Porosity-Free Alloy Steel by Application of High Oxygen-Containing Powders Mixed with Cr Particles. Vacuum.

[96] Li L. (2006). Repair of Directionally Solidified Superalloy GTD-111 by Laser-Engineered Net Shaping. J. Mater. Sci..

[97] AlShaer A.W., Li L., Mistry A. (2014). The Effects of Short Pulse Laser Surface Cleaning on Porosity Formation and Reduction in Laser Welding of Aluminium Alloy for Automotive Component Manufacture. Opt. Laser Technol..

[98] Gao W., Zhao S., Wang Y., Liu F., Zhou C., Lin X. (2014). Effect of Re-Melting on the Cladding Coating of Fe-based Composite Powder. Mater. Des..

[99] Zhou J., Tsai H. (2007). Effects of Electromagnetic Force on Melt Flow and Porosity Prevention in Pulsed Laser Keyhole Welding. Int. J. Heat Mass Transf..

[100] Zhang N., Liu W., Deng D., Tang Z., Liu X., Yan Z., Zhang H. (2018). Effect of Electric-Magnetic Compound Field on the Pore Distribution in Laser Cladding Process. Opt. Laser Technol..

[101] Wang D., Liang E., Chao M., Yuan B. (2008). Investigation on the Microstructure and Cracking Susceptibility of Laser-Clad V_2_O_5_ /NiCrBSiC Alloy Coatings. Surf. Coat. Technol..

[102] Cloots M., Uggowitzer P.J., Wegener K. (2016). Investigations on the Microstructure and Crack Formation of IN738LC Samples Processed by Selective Laser Melting Using Gaussian and Doughnut Profiles. Mater. Des..

[103] Bidron G., Doghri A., Malot T., Fournier-Dit-Chabert F., Thomas M., Peyre P. (2020). Reduction of the Hot Cracking Sensitivity of CM-247LC Superalloy Processed by Laser Cladding Using Induction Preheating. J. Mater. Process. Technol..

[104] Alizadeh-Sh M., Marashi S.P.H., Ranjbarnodeh E., Shoja-Razavi R. (2020). Laser Cladding of Inconel 718 Powder on a Non-Weldable Substrate: Clad Bead Geometry-Solidification Cracking Relationship. J. Manuf. Process..

[105] Ebrahimzadeh H., Farhangi H., Mousavi S.A. (2020). Hot Cracking in Autogenous Welding of 6061-T6 Aluminum Alloy by Rectangular Pulsed Nd:YAG Laser Beam. Weld. World.

[106] Näkki J., Tuominen J., Vuoristo P. (2017). Effect of Minor Elements on Solidification Cracking and Dilution of Alloy 625 Powders in Laser Cladding. J. Laser Appl..

[107] Bielenin M., Schmidt L., Schricker K., Bergmann J.P. (2019). Prevention of Solidification Cracking by Use of a Diode Laser Superposition in Pulsed Laser Beam Welding. Proc. SPIE.

[108] Wei H., Chen J.S., Wang H., Carlson B.E. (2016). Thermomechanical Numerical Analysis of Hot Cracking During Laser Welding of 6XXX Aluminum Alloys. J. Laser Appl..

[109] Opprecht M., Garandet J.-P., Roux G., Flament C., Soulier M. (2020). Solution to the Hot Cracking Problem for Aluminium Alloys Manufactured by Laser Beam Melting. Acta Mater..

[110] Chen L., Zhang X., Wu Y., Chen C., Li Y., Zhou W., Ren X. (2022). Effect of surface morphology and microstructure on the hot corrosion behavior of TiC/IN625 coatings prepared by extreme high-speed laser cladding. Corros. Sci..

[111] Yan H., Liu K., Zhang P., Zhao J., Qin Y., Lu Q., Yu Z. (2020). Fabrication and Tribological Behaviors of Ti3SiC2/Ti5Si3/TiC/Ni-based Composite Coatings by Laser Cladding for Self-Lubricating Applications. Opt. Laser Technol..

[112] Xu G., Kutsuna M., Liu Z., Zhang H. (2006). Characteristics of Ni-based Coating Layer Formed by Laser and Plasma Cladding Processes. Mater. Sci. Eng. A.

[113] Zhu L., Xue P., Lan Q., Meng G., Ren Y., Yang Z., Xu P., Liu Z. (2021). Recent research and development status of laser cladding: A review. Opt. Laser Technol..

[114] Wu X., Yan H., Xin Y., Baobiao Y., Zhi H., Yonghui S. (2020). Microstructure and Wear Properties of Ni-Based Composite Coatings on Aluminum Alloy Prepared by Laser Cladding. Rare Met. Mater. Eng..

[115] Chen C.J., Cao Q., Zhang M., Chang Q.M., Zhang S.C. (2011). Laser Repair Cladding of Ni-Base Alloy on TC2 Ti Alloy. Adv. Mater. Res..

[116] Wang Y., Liu N., Zhu F. (2011). Microstructure and Wear Resistance of Laser Cladding of Ni-based Alloy on Copper Substrate. Mater. Sci. Forum.

[117] Shan B., Chen J., Chen S., Ma M., Ni L., Shang F., Zhou L. (2022). Laser cladding of Fe-based corrosion and wear-resistant alloy: Genetic design, microstructure, and properties. Surf. Coat. Technol..

[118] Wan M.Q., Shi J., Lei L., Cui Z.Y., Wang H.L., Wang X. (2018). Comparative Study of the Microstructure, Mechanical Properties and Corrosion Resistance of Ni- or Fe- Based Composite Coatings by Laser Cladding. J. Mater. Eng. Perform..

[119] Chen J., Guo C., Zhou J. (2012). Microstructure and Tribological Properties of Laser Cladding Fe-based Coating on Pure Ti Substrate. Trans. Nonferr. Met. Soc. China.

[120] Jiang L., Cui X., Jin G., Tian H., Tian Z., Zhang X., Wan S. (2022). Synthesis and microstructure, properties characterization of Ni-Ti-Cu/Cu-Al functionally graded coating on Mg-Li alloy by laser cladding. Appl. Surf. Sci..

[121] Ye H., Peng S., Yan Z., Zhang X.B. (2013). Microstructure and Properties of Laser Cladding Fe-Al Intermetallics. Adv. Mater. Res..

[122] Bai H., Zhong L., Kang L., Liu J., Zhuang W., Lv Z., Xu Y. (2021). A review on wear-resistant coating with high hardness and high toughness on the surface of titanium alloy. J. Alloys Compd..

[123] Lu S., Wang L., Zhou J., Liang J. (2022). Microstructure and tribological properties of laser-cladded Co-Ti_3_SiC_2_ coating with Ni-based interlayer on copper alloy. Tribol. Int..

[124] Liu Y., Wang H.M. (2010). Microstructure and Wear Property of Laser-Clad Co_3_Mo_2_Si/Coss Wear Resistant Coatings. Surf. Coat. Technol..

[125] Guo J., Yan H., Zhang P., Yu Z., Lu Q., Chen Z. (2019). Laser cladding NiCrBSi/TiN/h-BN self-lubricating wear resistant coating on Ti–6Al–4V surface. Mater. Res. Express.

[126] Zhang X., Yocom C.J., Mao B., Liao Y. (2019). Microstructure evolution during selective laser melting of metallic materials: A review. J. Laser Appl..

[127] Chi Y., Gong G., Zhao L., Yu H., Tian H., Du X., Chen C. (2021). In-situ TiB2-TiC reinforced Fe-Al composite coating on 6061 aluminum alloy by laser surface modification. J. Mater. Process. Technol..

[128] Zhang P., Li Z., Liu H., Zhang Y., Li H., Shi C., Liu P., Yan D. (2022). Recent progress on the microstructure and properties of high entropy alloy coatings prepared by laser processing technology: A review. J. Manuf. Process..

[129] Zhang P., Liu X., Lu Y., Yan H., Yu Z., Li C., Lu Q. (2014). Microstructure and Wear Behavior of Cu–Mo–Si Coatings by Laser Cladding. Appl. Surf. Sci..

[130] Zhang H., Zou Y., Zou Z., Wu D. (2015). Microstructure and Properties of Fe-based Composite Coating by Laser Cladding Fe–Ti–V–Cr–C–CeO_2_ Powder. Opt. Laser Technol..

[131] Weng F., Yu H., Liu J., Chen C., Dai J., Zhao Z. (2017). Microstructure and Wear Property of the Ti_5_Si_3_ /TiC Reinforced Co-based Coatings Fabricated by Laser Cladding on Ti-6Al-4V. Opt. Laser Technol..

[132] Zhao Y., Yu T., Guan C., Sun J., Tan X. (2019). Microstructure and Friction Coefficient of Ceramic (TiC, TiN and B4C) Reinforced Ni-based Coating by Laser Cladding. Ceram. Int..

[133] Zhao T., Zhang S., Wang Z.Y., Zhang C.H., Liu Y., Wu C.L. (2022). Modulating heat input to optimize corrosion and synergistic cavitation erosion-corrosion behavior of Ni201 cladding layer by cold metal transfer. Surf. Coat. Technol..

[134] Chiu K., Cheng F., Man H. (2005). Laser cladding of austenitic stainless steel using NiTi strips for resisting cavitation erosion. Mater. Sci. Eng. A.

[135] Cheng F., Kwok C., Man H. (2002). Cavitation erosion resistance of stainless steel laser-clad with WC-reinforced MMC. Mater. Lett..

[136] Gonzalez-Parra J.C., Robles V., Devia-Cruz L.F., Rodriguez-Beltran R.I., Cuando-Espitia N., Camacho-Lopez S., Aguilar G. (2022). Mitigation of cavitation erosion using laser-induced periodic surface structures. Surf. Interface.

[137] Duraiselvam M., Galun R., Wesling V., Mordike B.L., Reiter R., Oligmüller J. (2006). Cavitation erosion resistance of AISI 420 martensitic stainless steel laser-clad with nickel aluminide intermetallic composites and matrix composites with TiC reinforcement. Suface Coat. Technol..

[138] Liu Y., Yang L., Yang X., Zhang T., Sun R. (2021). Optimization of microstructure and properties of composite coatings by laser cladding on titanium alloy. Ceram. Int..

[139] Chen S., Liang J., Liu C., Sun K., Mazumder J. (2011). Preparation of a Novel Ni/Co-based Alloy Gradient Coating on Surface of the Crystallizer Copper Alloy by Laser. Appl. Surf. Sci..

[140] Wang X., Zhang Z., Men Y., Li X., Liang Y., Ren L. (2020). Fabrication of nano-TiC Functional Gradient Wear-Resistant Composite Coating On 40Cr Gear Steel Using Laser Cladding Under Starved Lubrication Conditions. Opt. Laser Technol..

[141] Su Y., Chen B., Tan C., Song X., Feng J. (2020). Influence of Composition Gradient Variation on the Microstructure and Mechanical Properties of 316 L/Inconel718 Functionally Graded Material Fabricated by Laser Additive Manufacturing. J. Mater. Process. Technol..

[142] Yin T., Zhang S., Wang Z., Zhang C., Liu Y., Chen J. (2022). Effect of laser energy density on microstructural evolution and wear resistance of modified aluminum bronze coatings fabricated by laser cladding. Mater. Chem. Phys..

[143] LiuLiu Y., Ding Y., Yang L., Sun R., Zhang T., Yang X. (2021). Research and progress of laser cladding on engineering alloys: A review. J. Manuf. Process..

[144] Jiang X., Tian Z., Liu W., Tian G., Gao Y., Xing F., Suo Y., Song B. (2022). An energy-efficient method of laser remanufacturing process. Sustain. Energy Technol. Assess..

